# Polyyne-producing *Burkholderia* suppress *Globisporangium ultimum* damping-off disease of *Pisum sativum* (pea)

**DOI:** 10.3389/fmicb.2023.1240206

**Published:** 2023-08-25

**Authors:** Gordon Webster, Alex J. Mullins, Yoana D. Petrova, Eshwar Mahenthiralingam

**Affiliations:** Microbiomes, Microbes and Informatics Group, Organisms and Environment Division, School of Biosciences, Cardiff University, Cardiff, United Kingdom

**Keywords:** *Burkholderia*, polyynes, biopesticide, plant pathogens, biocontrol, *Pythium*

## Abstract

Extensive crop losses are caused by oomycete and fungal damping-off diseases. Agriculture relies heavily on chemical pesticides to control disease, but due to safety concerns multiple agents have been withdrawn. *Burkholderia* were successfully used as commercial biopesticides because of their fungicidal activity and plant protective traits. However, their potential for opportunistic pathogenicity led to a moratorium on their registration as biopesticides. Subsequently, *Burkholderia* were shown to produce multiple specialised metabolites including potent antimicrobial polyynes. Cepacin A, a polyyne produced by *Burkholderia ambifaria* biopesticide strains was shown to be an important metabolite for the protection of germinating peas against *Globisporangium ultimum* (formerly *Pythium*) damping-off disease. Recently, there has been an expansion in bacterial polyyne discovery, with the metabolites and their biosynthetic gene pathways found in several bacterial genera including *Burkholderia*, *Collimonas*, *Trinickia*, and *Pseudomonas*. To define the efficacy of these bacterial polyyne producers as biopesticidal agents, we systematically evaluated metabolite production, *in vitro* microbial antagonism, and *G. ultimum* biocontrol across a panel of 30 strains representing four bacterial genera. *In vitro* polyyne production and antimicrobial activity was demonstrated for most strains, but only *Burkholderia* polyyne producers were protective within the *in vivo G. ultimum* damping-off pea protection model. *B. ambifaria* was the most effective cepacin-expressing biopesticide, and despite their known potential for plant pathogenicity *Burkholderia gladioli* and *Burkholderia plantarii* were uniquely shown to be protective as caryoynencin-producing biopesticides. In summary, *Burkholderia* are effective biopesticides due to their suite of antimicrobials, but the ability to deploy polyyne metabolites, caryoynencin and cepacin, is strain and species dependent.

**Graphical Abstract:**
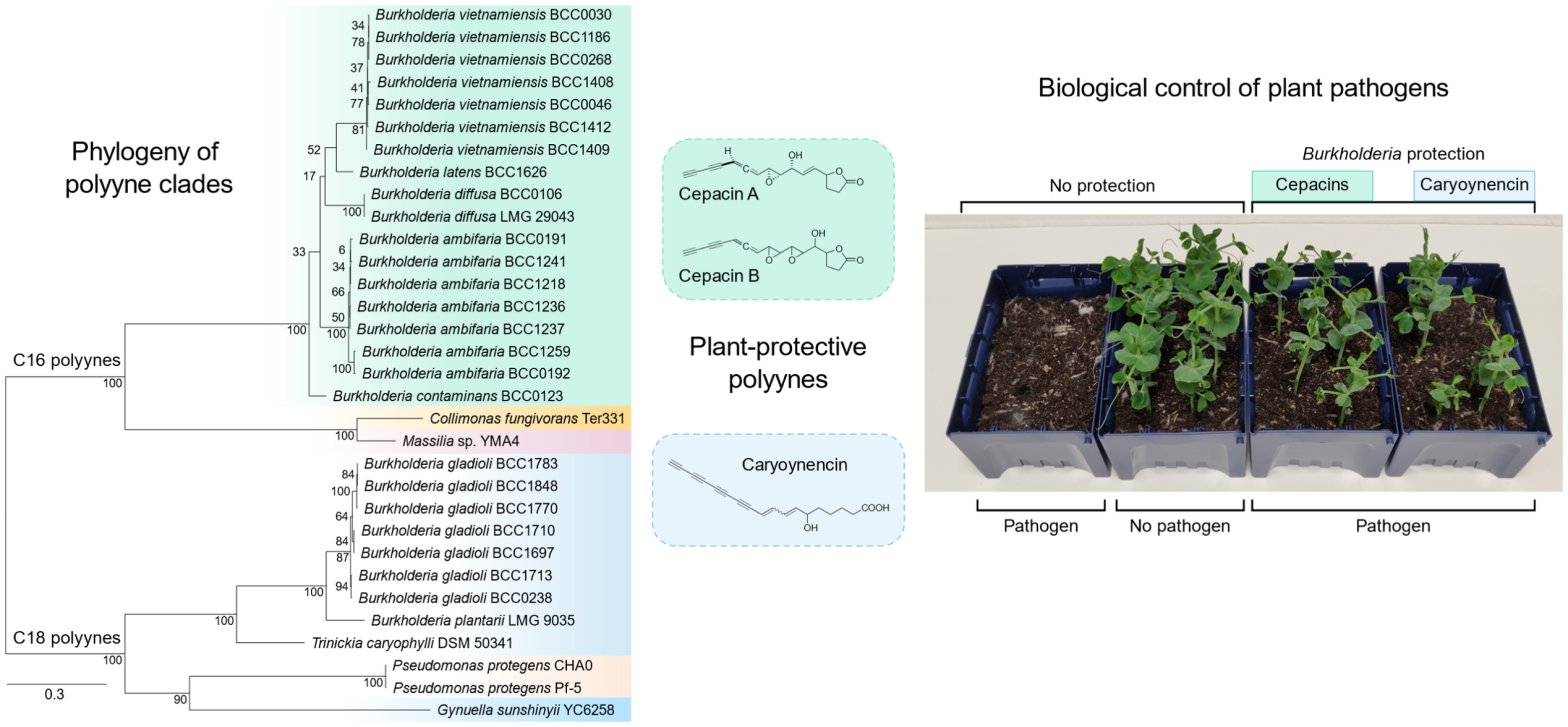

## 1. Introduction

Damping-off diseases that kill planted seeds and germinating crops are a global agricultural problem with an urgent need for new, sustainable control measures (Lamichhane et al., 2017). The oomycete *Globisporangium ultimum* Trow (synonym *Pythium ultimum* Trow; Uzuhashi et al., 2010) is a member of the *Pythium sensu* lato complex and causes damping-off and root-rot disease in a diverse range of plants, including *Pisum sativum* and other agriculturally important legumes (Hendrix and Campbell, 1973). Typically, infection occurs through mycelia or oospores persisting within the soil which then infect seeds and the root system leading to severe wilting, reduced yields, and plant death. This ultimately causes major global economic losses, notably in organic vegetable production (Alcala et al., 2016; Lamichhane et al., 2017). Commercial losses occur as direct costs from damage to the seeds and seedlings, or as indirect costs due to additional replanting and lower yields from delayed sowing times (Lamichhane et al., 2017).

Management of *G. ultimum* damping-off disease has mainly relied on the application of chemical pesticides, through soil fumigation or soil drenches; practises that are now being withdrawn due to their detrimental effects on the environment, human health, and the development of pesticide-resistant strains (Nicolopoulou-Stamati et al., 2016; Lamichhane et al., 2017). Therefore, finding novel disease management strategies and agents with less impact on the environment is of major importance, and also required to meet international objectives for environmental sustainability (Hulot and Hiller, 2021). Biological control of damping-off disease using naturally occurring antagonistic bacteria (biopesticides) is one such approach. The environmentally friendly potential of microbial biopesticides has led to a renewed interest in these disease control approaches (Fira et al., 2018; Mullins et al., 2019; Kumar et al., 2021). Several rhizosphere-colonising bacterial genera are capable of protecting their host plant from damping-off caused by *Pythium sensu* lato, including *Pseudomonas* (Gravel et al., 2005), *Streptomyces* (Punja and Yip, 2003), *Bacillus* (Fira et al., 2018), *Pantoea* (Bardin et al., 2003), and *Burkholderia* (Mao et al., 1997; Parke and Gurian-Sherman, 2001). Efficacy of biological control of damping-off has been demonstrated for several important crop species at a variety of scales, from laboratory models to commercial agricultural use (Lamichhane et al., 2017).

**Figure 1:**
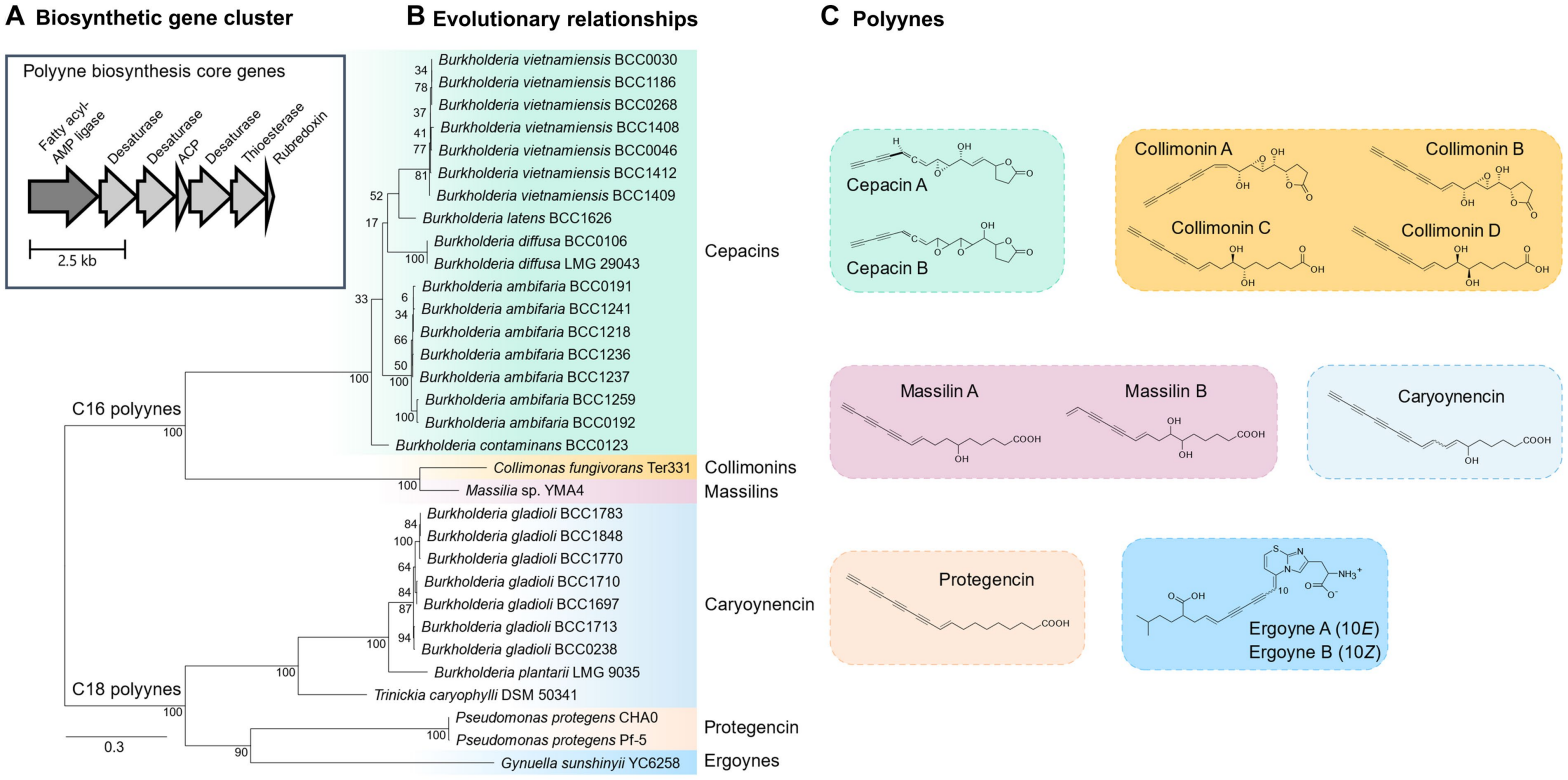
The core biosynthetic gene cluster, evolutionary relationships and polyynes produced by the bacteria evaluated for their ability to control damping-off disease. **(A)** The insert shows the seven essential genes for polyyne production present in all strains examined. **(B)** A phylogenetic tree based on the amino acid sequence of the fatty acyl-AMP ligase, one of the core genes responsible for polyyne biosynthesis (see inset) is presented to show the evolutionary diversity of the strains evaluated in this study. **(C)** The chemical structure of bacterial polyynes produced by each strain. Phylogenetic clades correspond to polyyne metabolites displayed with colour coding.

Recently, it was demonstrated that historically effective *Burkholderia* biopesticides (Parke and Gurian-Sherman, 2001), specifically the species *Burkholderia ambifaria* protected *Pisum sativum* seedlings against damping-off disease by *G. ultimum* through the production of the antimicrobial polyyne metabolite, cepacin A (Mullins et al., 2019). Polyynes are compounds with alternating triple and single carbon–carbon bonds that have attracted considerable interest because of their unusual structure, high reactivity, and antimicrobial properties (Ross et al., 2014). Multiple bacterial polyynes (Figure 1) have been discovered. They have been shown to have potent biological activities, including the antibacterial and antioomycete properties of cepacins (Parker et al., 1984; Mullins et al., 2019), the antibacterial activity of caryoynencin from *Trinickia caryophylli* (formerly *Burkholderia caryophylli*) (Kusumi et al., 1987; Ross et al., 2014), the antifungal properties of collimonins and massilins from *Collimonas fungivorans* and *Massilia* sp. YMA4, respectively (Fritsche et al., 2014; Kai et al., 2018; Lin et al., 2022), and the algicidal (Hotter et al., 2021) and antioomycete (Murata et al., 2021) activities of protegencin from *Pseudomonas protegens* (Mullins et al., 2021). Additionally, caryoynencin, together with other antimicrobial *Burkholderia gladioli* metabolites, has also been implicated in the ecological role of safe-guarding Lagriinae beetle eggs from attack by fungal pathogens (Flórez et al., 2017).

*Burkholderia* bacteria therefore represent a key group of polyyne producers and had been historically harnessed as commercial biopesticides because of their antimicrobial (Bach et al., 2021) and biological control activities (Parke and Gurian-Sherman, 2001). However, they are also human and plant pathogens, and in the absence of further understanding of their safety and bioactive mechanism, these concerns have restricted their exploitation as biocontrol agents (Eberl and Vandamme, 2016). We explored the capacity of *Burkholderia* and other polyyne-producing bacteria to protect *Pisum sativum* (peas) from damping-off disease caused by *G. ultimum*. Polyynes are inherently unstable and light-sensitive (Ross et al., 2014; Kai et al., 2018; Petrova et al., 2022) and direct delivery to the rhizosphere via bacterial seedcoats is required for biopesticidal efficacy (Mullins et al., 2019). A panel of 30 bacteria shown to encode a polyyne biosynthetic gene cluster (BGC) by genome mining (Figure 1; Mullins et al., 2021) was assembled and comprised of *Burkholderia* (7 species, 26 strains), *Pseudomonas protegens* (2 strains), *Trinickia caryophylli and Collimonas fungivorans* (1 strain each). The collection was assessed for antagonistic activity through *in vitro* antimicrobial assays, the production of polyynes and other compounds by metabolite analyses. The strains were subsequently tested within a *Pisum sativum* biological control assay for *G. ultimum* damping-off. The study expands the evidence that cepacin A production by some *B. ambifaria* strains protects pea plants from *G. ultimum* (Mullins et al., 2019), and uniquely highlights that other polyynes, such as caryoynencin, can be harnessed for the same biological control function. We also uniquely demonstrate that *Burkholderia* species such as *B. gladioli* and *B. plantarii*, more associated with plant-pathogenic traits (Maeda et al., 2006; Jones et al., 2021), initially form protective associations with germinating plants, in contrast to the opportunistic disease they may elicit on mature or damaged hosts.

## 2. Materials and methods

### 2.1. Strains and growth conditions

Strains of polyyne-producing bacteria used in this study (Figure 1 and Table 1) were obtained from the Cardiff University *Burkholderia* culture collection (Mullins et al., 2020) and other recognised strain repositories [The Belgium Co-ordinated Collections of Microorganisms/Laboratorium voor Microbiologie, Universiteit Gent (BCCM/LMG); The *Burkholderia cepacia* Research Laboratory and Repository (BcRLR; Lipuma, 2010); Leibniz Institute DSMZ-German Collection of Microorganisms and Cell Cultures GmbH (DSMZ)] and stored at −80°C in Tryptone Soya Broth (TSB; Oxoid™) containing 8% (v/v) dimethylsulfoxide (DMSO; Sigma-Aldrich). Cultures were revived onto Tryptone Soya Agar (TSA; Oxoid™) in Petri dishes and incubated at 30°C for 24 h. Bacterial cultures were routinely streaked to single colonies on TSA to check for purity. Overnight liquid cultures were prepared by inoculating 5 mL of TSB with confluent growth from a fresh TSA plate, incubated at 30°C on a rocking platform (150 rpm) and used as bacterial inoculum in specialised metabolite induction, *in vitro* antagonism, and biocontrol assays.

**Table 1:** Bacterial strains used in this study with their known polyyne biosynthetic gene cluster (BGC) and other known specialised metabolites and BGCs.

Species	Strain	Alternative strain name(s)	Isolation source	Polyyne BGC	Polyyne detection on BSMG^a^	Polyyne detection on PEM^a^	Other known specialised metabolites or BGCs^b^^,^^c^	Reference(s)^d^
*Burkholderia ambifaria*	BCC0191	HI2345; J82; R-5140; ATCC 51993	Soil, USA	cepacin	+	+	**pyrrolnitrin**, burkholdines, phenazine	Parke and Gurian-Sherman (2001) and Mullins et al. (2019)
	BCC0192	HI2347; Ral-3; R-8863	Maize rhizosphere, USA	cepacin	−	−	**pyrrolnitrin**, AFC-BC11, bactobolins, hydroxyquinolines	Coenye et al. (2001) and Mullins et al. (2019)
	BCC1218	MW80-16	Rhizosphere, USA	cepacin	+	+	**pyrrolnitrin**, burkholdines	Mullins et al. (2019)
	BCC1236	KC5-54	Maize rhizosphere, USA	cepacin	+	+	**pyrrolnitrin**, burkholdines	Mullins et al. (2019)
	BCC1237	KC10-16	Maize rhizosphere, USA	cepacin	+	+	**pyrrolnitrin**, burkholdines	Mullins et al. (2019)
	BCC1241	KC311-6	Maize rhizosphere, USA	cepacin	+	+	**pyrrolnitrin**, burkholdines	Mullins et al. (2019)
	BCC1259	KW20-2	Maize rhizosphere, USA	cepacin	+	+	**pyrrolnitrin**, hydroxyquinolines	Mullins et al. (2019)
*Burkholderia vietnamiensis*	BCC0030	LMG 10929^T^; FC0369	Rice rhizosphere, Vietnam	cepacin	+	+	−	Gillis et al. (1995); This study
	BCC0046	J1738	Patient wound, USA	cepacin	+	+	−	Baldwin et al. (2007); This study
	BCC0268	BBG1222	Soil, New Zealand	cepacin	+	+	−	Mullins et al. (2020); This study
	BCC1408	JW13.1a	Diesel contaminant, UK	cepacin	+	+	−	White et al. (2011); This study
	BCC1409	JW13.2a	Diesel contaminant, UK	cepacin	+	+	−	White et al. (2011); This study
	BCC1186	D1389	*CF* patient	cepacin	−	−	−	Jassem et al. (2011); This study
	BCC1412	JW14.1a	Diesel contaminant, UK	cepacin	+	+	−	White et al. (2011); This study
*Burkholderia diffusa*	LMG 29043	ATCC 39356; SC 11783	Soil, USA	cepacin	+	+	−	Parker et al. (1984); This study
	BCC0106	GJ; R-9912; CEP0472; LMG 24266	*CF* patient, Canada	cepacin	+	+	−	Vanlaere et al. (2008); This study
*Burkholderia latens*	BCC1626	LMG 24264	*CF* patient, UK	cepacin	+	+	−	Vanlaere et al. (2008); This study
*Burkholderia contaminans*	BCC0123	HW; CEP0624	*CF* patient sputum, USA	cepacin	+	−	**pyrrolnitrin**	Mullins et al. (2020); This study
*Burkholderia gladioli*	BCC0238	MA4	*CF* patient sputum, USA	caryoynencin	+	+	**toxoflavin, gladiolin**, icosolides	Song et al. (2017), Jones et al. (2021), and Jenner et al. (2019)
	BCC1697	AU18435	*CF* patient sputum, USA	caryoynencin	+	+	**toxoflavin, bongkrekic acid**, icosolides	Jones et al. (2021)
	BCC1710	AU21299	*CF* patient sputum, USA	caryoynencin	+	+	**toxoflavin, enacyloxin IIa, bongkrekic acid**, icosolides	Jones et al. (2021)
	BCC1713	AU21396	*CF* patient sputum, USA	caryoynencin	+	+	**toxoflavin, gladiolin**, icosolides	Jones et al. (2021)
	BCC1770	AU3822	*CF* patient sputum, USA	caryoynencin	+	+	**toxoflavin**, icosolides	Jones et al. (2021)
	BCC1883	−	−	caryoynencin	+	+	**toxoflavin, sinapigladioside, enacyloxin IIa**, icosolides	Jones et al. (2021)
	BCC1848	AU29552	*CF* patient sputum, USA	caryoynencin	+	+	**toxoflavin**, icosolides	Jones et al. (2021)
*Burkholderia plantarii*	BCC0777	LMG 9035^T^; ATCC 43733	Rice seedling with blight, Japan	caryoynencin	+	+	**tropolone**, iminopyrrolidines	Azegami et al. (1987), Kunakom and Eustáquio (2019); This study
*Trinickia caryophylli*	BCC0769	LMG 2155^T^; ATCC 25418	*Dianthus Caryophyllus*, USA	caryoynencin	+	+	trinickiabactin	Ross et al. (2014), Jiao et al. (2020), and Burkholder (1942)
*Collimonas fungivorans*	Ter331	LMG 21588	Dune soil, The Netherlands	collimonin	−	−	−	Fritsche et al. (2014), Kai et al. (2018), and De Boer et al. (2004)
*Pseudomonas protegens*	CHA0^T^	DSM 19095^T^	Tobacco roots, Switzerland	protegencin	+	+	**toxoflavin, pyoluteorin, 2,4-DAPG**, HCN, pyrrolnitrin	Neidig et al. (2011), Mullins et al. (2021), Stutz et al. (1986); This study
	Pf-5	ATCC BAA-477	Cotton rhizosphere, USA	protegencin	+	+	**toxoflavin, pyoluteorin, 2,4-DAPG**, HCN, pyoverdine, pyochelin, rhizoxin, pyrrolnitrin	Mullins et al. (2021), Howell and Stipanovic (1979), Paulsen et al. (2005), Loper et al. (2008), and Philmus et al. (2015); This study

a Polyynes detected by HPLC on agar solidified BSMG or PEM after growth at 22°C for 3 days.

b Known metabolites in strain or presence of a recognised biosynthetic gene cluster.

c Bold font indicates metabolites were detected by HPLC on BSMG or PEM after growth at 22°C for 3 days in this study.

d Reference(s) for strain isolation source and/or identification of known BGCs.

BSMG, basal salts medium with glycerol; PEM, pea seed exudate medium; 2,4-DAPG, 2,4-diacetylphloroglucinol; HCN, hydrogen cyanide.

Insertional mutants *Burkholderia ambifaria* BCC0191::*ccnJ* (Mullins et al., 2019) and *Burkholderia gladioli* BCC1697::*cayA* (Jones et al., 2021) with their respective fatty acyl-AMP ligase-encoding gene disrupted from the polyyne BGC were maintained as above, with the exception that 50 μg mL^−1^ trimethoprim was included in the media. *G. ultimum* var. *ultimum* MUCL 16164 was obtained from BCCM/MUCL (Mycothèque de l’Université Catholique de Louvain) collection and grown on potato dextrose agar (PDA; Oxoid™) plates at 22°C. For long term storage, cultures were maintained on PDA slants at 4°C.

### 2.2. Bioinformatic analysis

Polyyne BGC architecture figures were visualised using Clinker v0.0.21 (Gilchrist and Chooi, 2021), and metabolite structures created with ChemDraw Professional 16. BGC relatedness was then demonstrated by phylogenetic analysis. The amino acid sequence of the fatty acyl-AMP ligase present in every polyyne BGC was aligned using MAFFT v7.505 (Katoh and Standley, 2013), and a phylogenetic tree constructed with RAxML-NG v1.0.3 (Kozlov et al., 2019) using the LG + G8 + F model with 100 bootstraps.

### 2.3. Specialised metabolite induction media

Production of polyynes and other specialised metabolites were induced from bacteria by growing them at 22°C on two different media. Basal salts medium supplemented with glycerol (BSMG) as previously described (Mahenthiralingam et al., 2011; Webster et al., 2020a) and pea seed exudate medium (PEM; Mullins et al., 2021; Petrova et al., 2022). PEM was designed as a biomimetic medium to represent nutrient conditions during pea seed germination and was made as follows. Early Onward variety *Pisum sativum* seeds (approx. 100 g) were washed three times with deionised water, and then suspended in ultrapure water made up to 500 mL. Seeds were incubated in the dark with agitation (40 rpm on a rocking platform) for 2 d at 22°C. After incubation, seed exudate was removed and filtered twice. First with a Whatman® glass microfibre GF/D grade filter to remove seed coat material, and second with a Whatman® glass microfibre GF/A grade filter to obtain a clear seed exudate. The filtered seed exudate was diluted with ultrapure water at a 1:1 ratio and mixed with 1.5% (w/v) purified agar (Oxoid™) prior to autoclaving.

### 2.4. Specialised metabolite detection by HPLC

Detection of specialised metabolites was conducted according to the rapid screening method described previously (Webster et al., 2020a). In brief, bacterial strains were streaked onto 20 mL BSMG or PEM agar plates in duplicate, and incubated for 3 d at 22°C. Following incubation bacterial growth was removed from the agar surface, and a 20 mm diameter disc cut from the centre of the plate. The agar disc was placed into a 30 mL wide-mouth amber glass bottle with 0.5 mL dichloromethane and agitated on a rocking platform (40 rpm) for 2 h. Dichloromethane extracts were analysed by high performance liquid chromatography (HPLC) on a Waters® AutoPurification™ HPLC system fitted with a reversed-phase analytical column (Waters® XSelect CSH C18, 4.6 × 100 mm, 5 μm) and a C18 SecurityGuard™ cartridge (Phenomenex) in series. Detection of compounds was by absorbance at 210–400 by a photo-diode array detector (PDA). Mobile phases consisted of (A) water with 0.1% formic acid and (B) acetonitrile with 0.1% formic acid with a flow rate of 1.5 mL/min. Elution conditions were as follows: 0–1 min, 95% phase A/ 5% phase B; 1–9 min, gradient of phase A from 95 to 5% and gradient of phase B from 5 to 95%; 10 to 11 min, 5% phase A / 95% phase B; 11–15 min, 95% phase A / 5% phase B. Known specialised metabolites were identified by HPLC peak retention times and UV absorbance characteristics, and by referencing these to internal standards characterised by High Resolution Liquid Chromatography-Mass Spectrometry (LC–MS) and Nuclear Magnetic Resonance (NMR) as described (Mahenthiralingam et al., 2011; Song et al., 2017; Mullins et al., 2019; Webster et al., 2020a; Jones et al., 2021). Purified pyrrolnitrin (Sigma-Aldrich) and tropolone (Sigma-Aldrich) were used as additional standards to confirm HPLC detection and peak retention times for these specialised metabolites. Peak heights were calculated using MassLynx V4.1 software.^1^

### 2.5. *In vitro* microbial antagonism assays

Antagonism assays for polyyne-producing bacteria were performed against a panel of susceptibility organisms: *Pectobacterium carotovorum* LMG 2464 (Gram-negative bacterium), *Staphylococcus aureus* NCTC 12981 (Firmicutes Gram-positive bacterium), *Clavibacter michiganensis* DSM 46364 (Actinobacteria Gram-positive bacterium), and *Candida albicans* SC 5314 (fungus) as described (Mahenthiralingam et al., 2011; Mullins et al., 2019; Webster et al., 2020b). In brief, polyyne-producing bacterial strains were grown overnight at 30°C in TSB, spotted (2.0 μL bacteria) onto BSMG or PEM agar plates and incubated at 22°C for 3 d. Polyyne-producing bacteria were then killed by chloroform exposure for 3 min, overlaid with susceptibility organism-seeded (0.4% [v/v] bacteria) half-strength iso-sensitest agar (Oxoid™) and the overlay plate incubated at 30°C or 37°C for 24 h. The diameter of the inhibition zone was then measured through the centre of the polyyne-producing bacterium. The mean inhibition zone was calculated from two plates per treatment.

### 2.6. *In vitro Globisporangium ultimum* inhibition assay

Polyyne-producing bacterial strains were grown overnight at 30°C in TSB, spotted (5.0 μL bacteria) onto BSMG and PEM agar plates as four evenly spaced drops, allowed to dry and incubated at 22°C. After 24 h incubation, a 5.0 mm diameter plug of leading-edge growth of *G. ultimum* was placed in the centre of the four bacterial spots and the plates incubated again at 22°C for a further 48 h. The distance between the leading-edge of *G. ultimum* and the centre of each bacterial colony was measured, and the mean inhibition zone calculated for each treatment.

### 2.7. Biological control assays

*Globisporangium ultimum* infested soil (a non-sterile potting mix) was prepared as previously described (Mullins et al., 2019). Briefly, a fresh PDA plate (90 mm diameter) was inoculated with a 5.0 mm diameter PDA plug of *G. ultimum*, and incubated at 22°C for 3 d. The agar was cut into 1.0 cm^2^ squares, added to 120 g potting mix composed of 5-parts generic compost (Levington Advance Pot and Bedding M2; ICL UK) and 1-part sand (Horticultural Sharp Sand, Melcourt Industries Ltd), and incubated at 22°C for 3 d. The *G. ultimum* infested potting mix was combined with fresh potting mix to achieve a 0.25% (w/w) infested soil. *Pisum sativum* seeds were either planted in *G. ultimum* infested soil or control un-infested potting mix contained within recycled pipette tip boxes (Starlab (UK), Ltd.). Each treatment consisted of 16 pea seeds split between two boxes with 200 g potting mix per box.

Polyyne-producing bacterial seed coats were prepared by growing overnight liquid cultures in TSB at 30°C. Bacterial cultures were washed, resuspended, and concentrated in phosphate buffer solution (PBS). The concentrated bacterial suspension was diluted to allow optical density measurement on a spectrophotometer (Jenway 6300 Visible Spectrophotometer) and then adjusted to 5.0 OD_600_ nm, equivalent to approx. 1 × 10^9^ cfu (colony forming units) mL^−1^. Seeds were submerged in the bacterial suspension resulting in a standardised coating of approximately 10^7^ cfu ml^−1^ per seed (as demonstrated in Mullins et al., 2019), immediately planted, and watered with 30 mL deionised water. Plants were grown at 22°C in propagators to maintain high humidity and placed in a Fitotron® plant growth chamber set at 16:8 h photoperiod and 40% relative humidity for 14 d. All bacterial strains were assayed for biocontrol potential in a minimum of 2 experiments (at least 2 × 16 seeds per treatment), along with uninoculated seeds (PBS only) and *Burkholderia ambifaria* BCC0191 coated seeds as controls in every experiment. Treatments were assessed over 12 independent experiments (Table 2) due to restrictions in growth chamber capacity.

**Table 2:** Biocontrol experiments to test polyyne-producing bacteria efficacy to control damping-off disease of *Pisum sativum*.

Treatment^a^	Experiment number^b^													
	1	2	3	4	5	6	7	8	9	10	Mean ± SD (n)	11	12	Mean ± SD (n)
No treatment	100	100	93.8	100	100	100	100	100	100	100	99.4 ± 2.0 (10)	93.8	93.8	93.8 ± 0 (2)
*Globisporangium ultimum (Gu)*	6.3	0	6.3	0	0	0	0	0	0	0	1.3 ± 2.6 (10)	0	0	0 ± 0 (2)
*Gu* + *B. ambifaria* BCC0191	68.8	56.3	75.0	25.0	50.0	50.0	37.5	50.0	31.3	62.5	50.6 ± 16.0 (10)	56.3	75.0	65.6 ± 13.3 (2)
*Gu* + *B. ambifaria* BCC0192	–	–	18.8	–	–	–	–	–	–	12.5	15.6 ± 4.4 (2)	–	–	–
*Gu* + *B. ambifaria* BCC1218	–	–	25.0	–	–	–	–	–	–	25.0	25.0 ± 0 (2)	–	–	–
*Gu* + *B. ambifaria* BCC1236	–	–	0	–	–	–	–	–	–	6.3	3.1 ± 4.4 (2)	–	–	–
*Gu* + *B. ambifaria* BCC1237	–	–	12.5	–	–	–	–	–	–	12.5	12.5 ± 0 (2)	–	–	–
*Gu* + *B. ambifaria* BCC1241	43.8	–	37.5	–	–	–	–	–	–	25.0	35.4 ± 9.5 (3)	–	–	–
*Gu* + *B. ambifaria* BCC1259	–	–	6.3	–	–	–	–	–	–	6.3	6.3 ± 0 (2)	–	–	–
*Gu* + *B. vietnamiensis* BCC0030	–	–	–	0	–	–	–	0	–	–	0 ± 0 (2)	–	–	–
*Gu* + *B. vietnamiensis* BCC0046	–	–	–	0	–	–	–	0	–	–	0 ± 0 (2)	–	–	–
*Gu* + *B. vietnamiensis* BCC0268	–	0	–	0	0	–	–	0	–	–	0 ± 0 (4)	–	–	–
*Gu* + *B. vietnamiensis* BCC1186	–	–	–	0	–	–	–	0	–	–	0 ± 0 (2)	–	–	–
*Gu* + *B. vietnamiensis* BCC1408	–	–	–	0	–	–	–	0	–	–	0 ± 0 (2)	–	–	–
*Gu* + *B. vietnamiensis* BCC1409	–	–	–	6.3	–	–	–	0	–	–	3.1 ± 4.4 (2)	–	–	–
*Gu* + *B. vietnamiensis* BCC1412	–	–	–	0	–	–	–	6.3	–	–	3.1 ± 4.4 (2)	–	–	–
*Gu* + *B. contaminans* BCC0123	–	–	–	18.8	–	–	–	–	0	–	9.4 ± 13.3 (2)	–	–	–
*Gu* + *B. diffusa* LMG 29043	31.3	12.5	–	–	12.5	–	–	–	–	–	18.8 ± 10.8 (3)	–	–	–
*Gu* + *B. diffusa* BCC0106	–	–	–	–	12.5	6.3	–	–	6.3	–	8.3 ± 3.6 (3)	–	–	–
*Gu* + *B. gladioli* BCC0238	–	–	–	–	–	12.5	0.0	–	–	–	6.3 ± 8.8 (2)	–	–	–
*Gu* + *B. gladioli* BCC1697	–	–	56.3	–	75.0	68.8	50.0	–	–	43.8	58.8 ± 13.0 (5)	62.5	50.0	56.3 ± 8.8 (2)
*Gu* + *B. gladioli* BCC1710	–	–	–	–	–	37.5	50.0	–	–	–	43.8 ± 8.8 (2)	–	–	–
*Gu* + *B. gladioli* BCC1713	–	–	–	–	–	12.5	6.3	–	–	–	9.4 ± 4.4 (2)	–	–	–
*Gu* + *B. gladioli* BCC1770	–	–	–	–	–	43.8	25.0	–	–	–	34.4 ± 13.3 (2)	–	–	–
*Gu* + *B. gladioli* BCC1883	–	–	–	–	–	50.0	62.5	–	–	–	56.3 ± 8.8 (2)	–	–	–
*Gu* + *B. gladioli* BCC1848	–	–	–	–	–	12.5	0	–	–	–	6.3 ± 8.8 (2)	–	–	–
*Gu* + *T. caryophylli* BCC0769	–	0	–	–	0	–	–	–	–	–	0 ± 0 (2)	–	–	–
*Gu* + *B. plantarii* BCC0777	–	–	–	–	–	–	62.5	68.8	62.5	–	64.6 ± 3.6 (3)	–	–	–
*Gu* + *C. fungivorans* Ter331	0	–	–	–	0	–	–	–	–	–	0 ± 0 (2)	–	–	–
*Gu* + *P. protegens* Pf-5	–	6.25	–	–	–	–	6.3	–	0	–	4.2 ± 3.6 (3)	–	–	–
*Gu* + *P. protegens* CHA0	–	–	–	–	0	6.3	–	–	0	–	2.1 ± 3.6 (3)	–	–	–
*Gu* + *B. ambifaria* BCC191::*ccnJ*	–	–	–	–	–	–	–	–	–	–	–	31.3	31.3	31.3 ± 0 (2)
*Gu* + *B. gladioli* BCC1697::*cayA*	–	––	–	–	–	–	–	–	–	–	–	31.3	18.8	25.0 ± 8.8 (2)

a *Gu, Globisporangium ultimum* (formerly *Pythium ultimum*) causal agent of damping off disease in peas.

b Experiments 1–10 were used to compile data shown in Figure 3B. Experiments 11 and 12 were used to compile data shown in Supplementary Figure S3.

Means were calculated on data from different experiments after Levene’s test showed that the variances for protection between treatments (using BCC1697 and BCC0191) was not significantly different, *F*(1,12) = 0.28, *p* = 0.606.

### 2.8. *Burkholderia ambifaria* root colonisation and soil persistence assays

The ability of three *B. ambifaria* strains (BCC0191, BCC1237, and BCC1259) to colonise the root system of *P. sativum* was assessed. Seeds were coated with bacterial cell suspensions of approximately 1 × 10^9^ cfu mL^−1^ and planted in potting mix as described above. Following 7 d of growth, the seedlings were removed and the first 2 cm segment of root from the seed was excised. This root section was macerated with a pestle in a 1.5 mL Eppendorf tube with 1 mL PBS and serially diluted in PBS. Serial dilutions were plated onto *Burkholderia cepacia* selective agar (BCSA, Oxoid™) and incubated overnight at 37°C to determine the *B. ambifaria* cfu per root section.

To assess soil persistence, 10 μL of approximately 1 × 10^9^ cfu mL^−1^ of each *B. ambifaria* strain (BCC0191, BCC1237, and BCC1259) was added to 1 g of hydrated potting mix (50% water content) in 25 mL sterile universal bottles. Each treatment was replicated six times, and initially three replicates per strain were serially diluted in PBS to determine the recoverable cfu g^−1^ at day 0. The remaining inoculated potting mix samples were incubated at 22°C for 7 d. Following incubation each 1 g of potting mix was serially diluted in PBS and plated onto BCSA to calculate the remaining cfu g^−1^. Control samples (no added bacteria) were also set-up and treated as described above. No *Burkholderia* growth was identified on BCSA from all control samples.

### 2.9. Expression analysis of cepacin biosynthetic gene cluster during *Pisum sativum* colonisation

The relative expression of the cepacin BGC across three *B. ambifaria* strains (BCC0191, BCC1237, and BCC1259) was determined by reverse-transcriptase (RT)-PCR. *P. sativum* seeds were coated with *B. ambifaria* and grown in potting mix as described above or on Whatman® filter paper grade 1 soaked in ultrapure water. Following 3 d of growth the seedlings were removed and 1 cm of root was excised, macerated, and pooled from three seedlings per treatment. Total RNA was extracted from the pooled samples using the FastRNA™ Pro Soil-Direct Kit (MP Biomedicals) and DNase I (RNase-free; New England Biolabs) treated according to the manufacturer’s protocol. RT-PCR was performed using the OneTaq® One-Step RT-PCR Kit (New England Biolabs) according to manufacturer’s protocols. PCR primers were designed to target one of the polyyne-associated desaturase genes in the cepacin BGC [*ccnN*; (Mullins et al., 2019)] and amplify a 514 bp product: Fwd: 5’-CTG TTC TGG GCA GGT ACG TT-3′ and Rev.: 5′-TGT CGT AGA AGT GGC AGT GG-3′. Thermal cycler conditions for RT-PCR were as follows: RT at 48°C for 15 min; initial denaturation at 94°C for 1 min; 35x cycles of denaturation at 94°C for 15 s, annealing at 60°C for 30 s, and extension at 68°C for 30 s; then a final extension at 68°C for 5 min. RNA extracted from *B. ambifaria* BCC0191 grown on BSMG and molecular grade water (Severn Biotech Ltd.) were used as positive and negative controls, respectively.

## 3. Results and discussion

### 3.1. Polyyne-producing bacteria synthesise a suite of specialised metabolites *in vitro*

**Figure 2:**
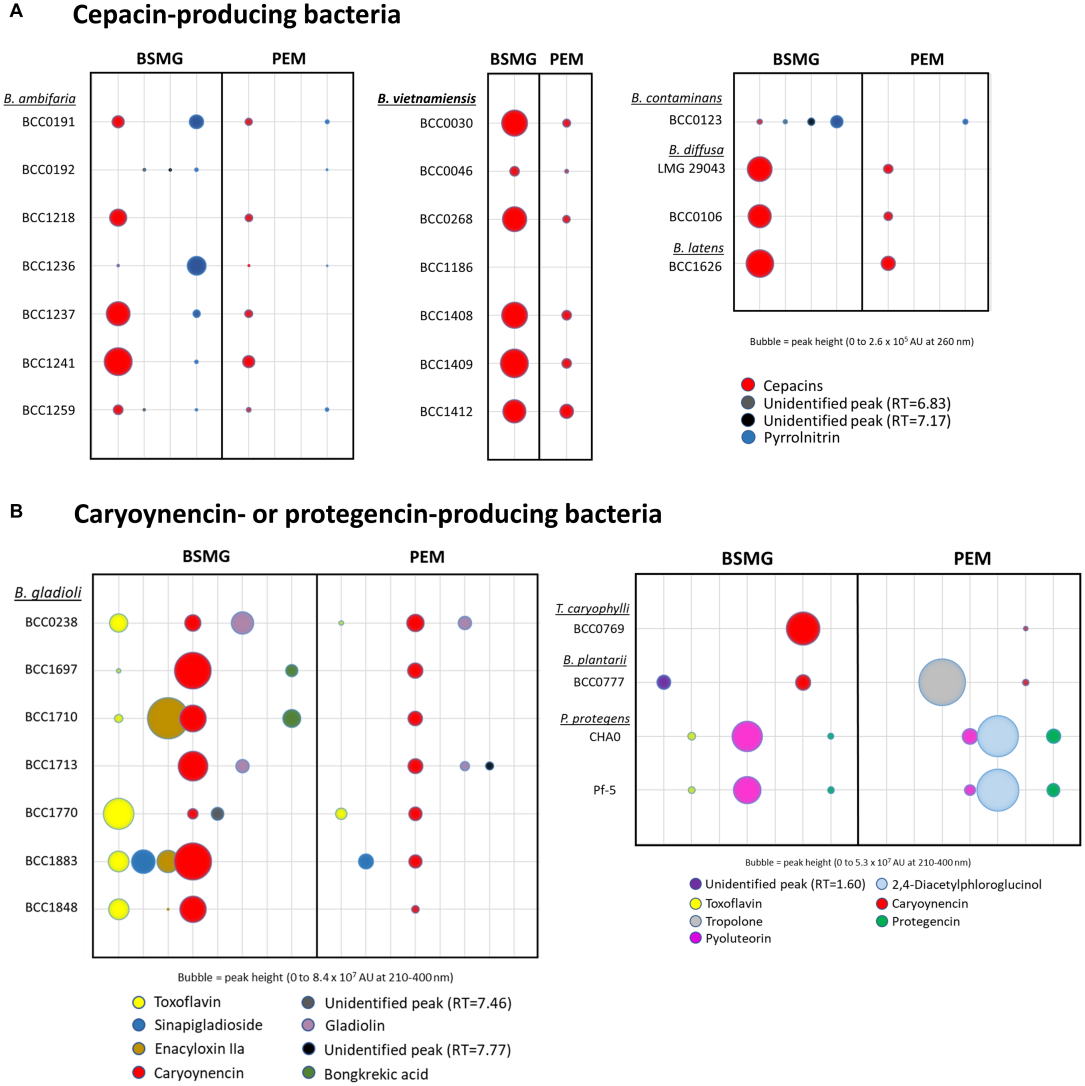
Detection and semi-quantitation of antimicrobial metabolites in polyyne-producing bacteria by HPLC. **(A)** Cepacin producing bacteria: *Burkholderia ambifaria*; *Burkholderia vietnamiensis*; *Burkholderia contaminans*; *Burkholderia diffusa*; *Burkholderia latens*. **(B)** Caryoynencin- and protegencin-producing bacteria: *Burkholderia gladioli*; *Trinickia caryophylli*; *Burkholderia plantarii*; *Pseudomonas protegens*. The size of bubble indicates relative peak height of metabolite observed on HPLC chromatograms (0–2.6 × 10^5^ AU at 260 nm; 0–5.3 × 10^7^ or 0–8.4 × 10^7^ AU at 210–400 nm). The colour of bubble correlates to a specific metabolite as shown on each key. Metabolite production was evaluated on BSMG (basal salt medium with glycerol) and PEM (pea exudate medium). Other abbreviations: RT = HPLC retention time (mins); AU = absorbance units measured at 260 nm **(A)** or 210–400 nm **(B)**.

A systematic collection of 30 bacteria that all encoded polyyne BGCs (Mullins et al., 2021) was assembled and comprised: seven *Burkholderia* species (26 strains), *Pseudomonas protegens* (2 strains), and one strain of *Trinickia caryophylli* and *Collimonas fungivorans* (Table 1). To evaluate their ability to produce polyynes (Figure 1) and other specialised metabolites *in vitro*, they were grown on metabolite induction media and subjected to chemical analysis by HPLC (Webster et al., 2020a). Specifically, metabolite production after growth on a minimal medium, with glycerol as a carbon source (BSMG) known to induce specialised metabolites in *Burkholderia* species (Mahenthiralingam et al., 2011; Webster et al., 2020a), was compared to that seen on the novel biomimetic medium made from the exudate of pea seeds (PEM; Mullins et al., 2021; Figure 2). Of the 18 bacterial strains carrying the cepacin BGC, 15 of these strains produced cepacin on BSMG and PEM, while *B. contaminans* BCC0123 only produced detectable levels of cepacin on BSMG (Figure 2A and Table 1). *B. ambifaria* BCC0192 and *B. vietnamiensis* BCC1186 did not produce detectable levels of cepacin on either medium. Cepacin production levels were universally greater on BSMG compared to PEM, although the differences in metabolite production between the two media for a given strain varied considerably (Figure 2A). The composition of PEM was analysed using liquid chromatography-mass spectrometry by Lifeasible.^2^ A large fraction of identified compounds (35%) contained a glycerol moiety (glycerophosphocholines, monoacylglycerides, glycerophosphoethanolamines, glycerophosphates, glycerophosphoserines, and triacylglycerols; Supplementary Figure S1), and suggests that natural concentrations of glycerol-containing compounds found in PEM are important to induce bacterial metabolites similar to the effect of glycerol in BSMG (Mahenthiralingam et al., 2011).

A similar trend of higher production on BSMG was also observed for the antifungal compound, pyrrolnitrin (El-Banna and Winkelmann, 1998) in *B. ambifaria* and *B. contaminans* BCC0123 (Figure 2A). Interestingly, strains *B. ambifaria* BCC0192 and *B. contaminans* BCC0123 also produced detectable levels of two other unknown metabolite peaks on BSMG (retention time (RT) = 6.83 min, UV absorbance = 323 & 336 nm; RT = 7.17 min, UV absorbance = 300 nm), and *B. ambifaria* BCC1259 produced only one of these extra HPLC peaks (RT = 6.83). It is possible that these peaks could be attributed to the presence of hydroxyquinolones and bactobolins, as *B. ambifaria* BCC0192 is known to have BGCs that encode for both these molecules, while *B. ambifaria* BCC1259 has only the hydroxyquinolone BGC (Mullins et al., 2019; Table 1).

A wider range of known specialised metabolites (toxoflavin, sinapigladioside, enacyloxin, caryoynencin, gladiolin and bongkrekic acid) were detected across the seven *B. gladioli* strains screened (Figure 2B). The production levels of the *B. gladioli* metabolites were also generally higher on BSMG than PEM (Figure 2B). The polyyne caryoynencin was detected in extracts from all seven *B. gladioli* strains and represented a universally induced and dominant metabolite when they were grown on BSMG and PEM (Figure 2B). Intriguingly, toxoflavin, a broad range phytotoxin and known antifungal compound (Furuya et al., 1997; Li et al., 2019) shown to be ubiquitous in *B. gladioli* (Webster et al., 2020a; Jones et al., 2021), was readily induced on BSMG, but absent in five strains, and present at very low levels in two strains when they were grown on germinating plant-mimicking PEM agar (Figure 2B). Similarly, the induction of the respiratory toxin bongkrekic acid (Anwar et al., 2017) and the antibiotic enacyloxin (Song et al., 2017; Jones et al., 2021) was also abrogated on the biomimetic PEM (Figure 2B). The antifungal isothiocyanate, sinapigladioside (Dose et al., 2021), from *B. gladioli* strain BCC1883 was induced on both BSMG and PEM. Two further uncharacterised *B. gladioli* metabolite peaks were also identified by HPLC and these displayed differential production on BSMG and PEM. One compound (RT = 7.46, UV absorbance = 281 nm) from *B. gladioli* BCC1770 was observed exclusively on BSMG, while the unknown compound (RT = 7.77, UV absorbance = 293, 308, 338, 363 & 392 nm) from *B. gladioli* BCC1713 was only observed on PEM (Figure 2B).

Specialised metabolites including polyynes were detected *in vitro* for *B. plantarii*, *T. caryophylli* and *P. protegens* on BSMG and PEM (Figure 2B), but no compounds including the polyynes, collimonins (Kai et al., 2018), were identified for *C. fungivorans* Ter331 (Table 1) under the conditions tested. Caryoynencin production by *B. plantarii* and *T. caryophylli* was induced at higher levels on BSMG than PEM (Figure 2B) in a similar manner to *B. gladioli*. *B. plantarii* also produced high levels of the antimicrobial compound, tropolone (Guo et al., 2019) on PEM, and an unidentified novel peak on BSMG (RT = 1.60, UV absorbance = 325 nm). Tropolone has been identified as a phytotoxin that causes bacterial seedling blight of rice caused by *B. plantarii* (Azegami et al., 1987).

In contrast to the polyynes isolated from *Burkholderia* and *Trinickia*, elevated production levels of the newly described polyyne protegencin from *P. protegens* (Mullins et al., 2021) were observed on PEM when compared to BSMG (Figure 2B). The well-characterised *P. protegens* specialised metabolites pyoluteorin and 2,4-diacetylphloroglucinol (2,4-DAPG) (Neidig et al., 2011) showed differential production for both strains CHA0 and Pf-5 as follows (Figure 2B). Pyoluteorin production was 3-5-fold higher on BSMG compared to PEM, while 2,4-DAPG was not produced on BSMG but only detected at high concentrations on PEM agar (Figure 2B). In parallel to the response of *B. gladioli*, toxoflavin production in *P. protegens* was also diminished on PEM. The suppression of broadly toxic compounds such as toxoflavin and bongkrekic acid by PEM are important considerations if polyyne-producing bacteria are to be explored for use as biopesticides. It was therefore particularly encouraging to note that cepacin, caryoynencin, and protegencin were consistently induced and toxic metabolite production was suppressed on PEM, which mimics the pea plant root system (Figure 2).

### 3.2. Broad *in vitro* antimicrobial activity of polyyne producing bacteria

**Figure 3:**
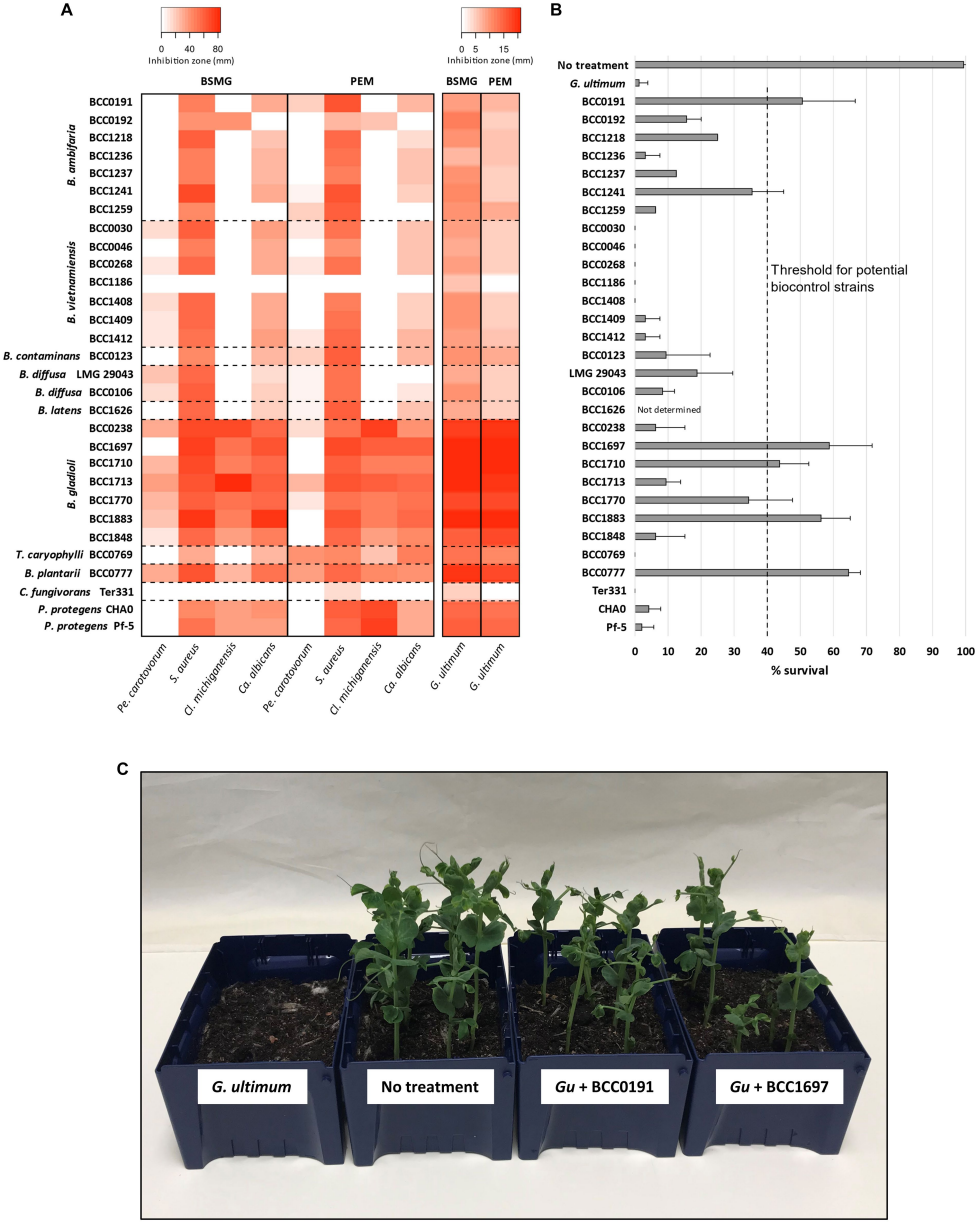
*In vitro* antimicrobial activity and *in vivo* biocontrol efficacy of bacteria carrying polyyne BGCs. **(A)** The bacteria possessing the cepacin, caryoynencin, collimonin, or protegencin biosynthetic gene clusters (*n* = 30 strains) were grown on BSMG and PEM agar. Microbial antagonism against the following pathogens was assessed: *Pectobacterium carotovorum*, *Staphylococcus aureus*, *Clavibacter michiganensis*, *Candida albicans*, and *Globisporangium ultimum*, and is presented as a heat-map. Zones of clearing (top left key) or contact inhibition (top right key) are indicated for each respective antagonism assay (see Methods). **(B)** Biological control efficacy of a seed coat of the polyyne encoding bacteria against *G. ultimum* is shown by the mean rate of survival of *Pisum sativum* seedlings after 14 d. **(C)** An example of one biological control plant assay with four different treatments shown as follows: *G. ultimum*; no treatment control; *G. ultimum* with *B. ambifaria* BCC0191; *G. ultimum* with *B. gladioli* BCC1697.

To complement the detection of specialised metabolites by HPLC we investigated the *in vitro* antimicrobial activity (Webster et al., 2020b) of polyyne producing bacteria, again comparing BSMG and PEM agar growth media. Antagonistic activity (Figure 3A and Supplementary Figure S2) was examined against a panel of plant and human pathogens comprising an oomycete (*G. ultimum*), a fungus (*Candida albicans*), a Gram-negative bacterium (*Pectobacterium carotovorum*) and two Gram-positive bacteria (*Staphylococcus aureus, Clavibacter michiganensis*). All polyyne-producing bacteria possessed activity against *G. ultimum* when grown on BSMG, and overall, antagonism on BSMG was greater compared to the activity on PEM (Figure 3A). Growth on BSMG also induced greater antimicrobial activity against *Ca. albicans* compared to PEM. In contrast, *S. aureus* antagonism was similar or higher when polyyne-producing bacteria were grown on PEM compared to BSMG (Figure 3A). Inhibition of *Pe. carotovorum* varied between media and polyyne-producing species. *B. ambifaria*, *B. contaminans* and *T. caryophylli* exhibited stronger antagonism of the Gram-negative plant pathogen when grown on PEM, while *B. vietnamiensis*, *B. diffusa* and *B. gladioli* possessed greater *Pe. carotovorum* antagonism on BSMG. *B. latens*, *C. fungivorans*, and *P. protegens* lacked detectable Gram-negative activity on both media. Bioactivity against *Cl. michiganensis* was observed for *B. gladioli*, *B. plantarii*, *P. protegens* and surprisingly only one strain of *B. ambifaria* (BCC0192) on both BSMG and PEM. Interestingly, the caryoynencin-producing *B. gladioli* and *B. plantarii* strains possessed the greatest antimicrobial activity against all five susceptibility test organisms (Figure 3A), specifically the three plant pathogens (*G. ultimum, Pe. carotovorum*, and *Cl. michiganensis*).

### 3.3. The ability of polyyne-producing bacteria to mediate biological control when applied as a seed coat is strain and species dependent

To understand the influence of different strains and/or species, and distinct polyynes on the biological control ability of *G. ultimum* damping-off disease, a comparison of the polyyne-producing strains representing nine species was performed (Figures 3B,C). Biocontrol efficacy was assessed using an *in vivo P. sativum* (pea) germination assay challenged with *G. ultimum* damping-off disease, as applied to evaluate *B. ambifaria* strains (Mullins et al., 2019). The strains evaluated carried the BGC for one of the four characterised polyynes: cepacin, caryoynencin, collimonins, or protegencin. As described above all the strains, except *C. fungivorans*, had been demonstrated to produce their respective polyynes (Figure 2 and Table 1) and possess antimicrobial activity (Figure 3A) *in vitro*. A plant survival rate of 40% was defined as an arbitrary threshold for successful biological control efficacy, as this minimum protection was consistently achieved by the prototypic biopesticide strain *B. ambifaria* BCC0191 (Mullins et al., 2019). Of the seven *B. ambifaria* strains examined, only BCC0191 achieved *P. sativum* protection with mean survival rates of 40% or higher. While biocontrol was observed in the remaining six *B. ambifaria* strains, the *P. sativum* survival rate they achieved ranged between 3.1 and 35.4% (Figure 3B and Table 2). Despite the close evolutionary relationship between *B. ambifaria* and *B. vietnamiensis* as members of the *B. cepacia* complex (Jin et al., 2020; Mullins and Mahenthiralingam, 2021) and the ability of 6 *B. vietnamiensis* strains to produce cepacin *in vitro* (Figure 2A), *B. vietnamiensis* produced essentially no biocontrol of *G. ulitimum* in the pea protection assay (Figure 3B). All seven *B. vietnamiensis* strains achieved between 0 and 3.1% pea mean pea survival rates (Figure 3B). Other cepacin-producing species, such as *Burkholderia diffusa* and *Burkholderia contaminans* were more efficacious than *B. vietnamiensis*, but still conferred less than 40% *P. sativum* mean survival rates (Figure 3B).

Caryoynencin-producing *B. gladioli* and *B. plantarii* strains were shown for the first time to be effective at protecting *P. sativum* seedlings from *G. ultimum*. Three of the seven *B. gladioli* strains (BCC1697, BCC1710, BCC1883) elicited mean survival rates of >40%, and strain BCC1697 achieved up to 75% (mean 58.8%) survival (Figure 3B and Table 2). Interestingly, despite producing lower levels of caryoynencin than the three effective strains *in vitro* on BSMG and PEM (Figure 2B), strain BCC1770 produced mean protection against damping-off disease *in vivo* that was just below the minimum threshold and therefore could also have potential as a biocontrol agent (Figure 3B and Table 2). *Burkholderia plantarii* BCC0777, a caryoynencin-producing strain, conferred a mean pea survival rate of 64.6%, similar to that of the best performing *B. gladioli* strains (Figure 3B). The high protection provided by *B. gladioli* and *B. plantarii* as a seed coat in this biocontrol model is significant since both species belong to the group of *Burkholderia* better known to cause disease symptoms in a range of plant species (Maeda et al., 2006; Jones et al., 2021). *B. gladioli* are well known as a causative agent of rot diseases in onions and mushrooms (Jones et al., 2021), and their protection of peas against *G. ultimum* suggests that these bacteria do not elicit these traits against a young, germinating plant.

The remaining strains examined in the *in vivo* biocontrol assay were selected because they encoded two further polyyne metabolites, the collimonins and protegencin. Despite evidence of *in vitro* production of protegencin from both *P. protegens* strains on PEM, neither of the strains demonstrated significant protection against *G. ultimum* (Figure 3B; mean pea survival rate < 4.2%). No collimonin production was detected from *C. fungivorans* Ter331 *in vitro* (Table 1) and no protection was observed in the biocontrol assay (Figure 3B).

Effective biological control of damping-off disease by *G. ultimum* was lost when either cepacin or caryoynencin production was abolished through the disruption of the polyyne BGC by insertional mutagenesis of the fatty acyl-ACP ligase gene. The *B. ambifaria* BCC0191::*ccnJ* cepacin mutant produced a mean pea survival rate of 31.3% (Table 2 and Supplementary Figure S3), corresponding to previous results observed for this biopesticidal strain (Mullins et al., 2019). Expanding on the finding that cepacin is a key anti-damping off protection agent (Mullins et al., 2019), the considerably reduced protection (25.0%, Table 2 and Supplementary Figure S3) elicited by the *B. gladioli* BCC1697::*cayA* mutant for the first time implicates that the polyyne caryoynencin can also offer biocontrol protection against *G. ultimum in vivo*. The plant protective effects of different polyynes have also been reported previously. For example, protegencin (alternatively named protegenin) from *Pseudomonas protegens* strain Cab57 (Murata et al., 2021) was attributed as a key bacterial metabolite in the protection of cucumber seedlings against *Pythium* damping-off disease. In addition, the antifungal and antioomycete properties of collimonins have also led them to being suggested as biocontrol agents for suppressing plant pathogens (Fritsche et al., 2014; Kai et al., 2018), although *in vivo* efficacy of *C. fungivorans* to protect peas against damping-off was not demonstrated in our study (Figure 3B). Furthermore, closely related *Paraburkholderia* species have been genetically engineered to express the polyyne BGCs, cepacin and caryoynencin (Petrova et al., 2022).

There is a history of *Burkholderia* species being used as commercial biopesticides with several *Burkholderia* strains being registered in the United States from 1992 and applied as soil treatments to control phytopathogenic fungi, damping-off and other plant diseases (Parke and Gurian-Sherman, 2001). Commercial biopesticide products were sold under the brand names Deny®, Blue Circle®, and Intercept® and contained mixtures of three *Burkholderia* strains, M36, M54 and J82 (Parke and Gurian-Sherman, 2001). However, these products were eventually withdrawn from the market after a 1999 US Environmental Protection Agency (EPA) risk assessment that resulted in a moratorium being issued on the registration of new biopesticidal products containing members of the *Burkholderia cepacia* complex or any bacteria with “affinities to a human opportunistic pathogen” until they could be proven safe (Parke and Gurian-Sherman, 2001). Biopesticide M36 was subsequently found to be a *B. cenocepacia* strain, while strains M54 and J82 (the original strain name of BCC0191 characterised herein; Mullins et al., 2019), were both *B. ambifaria* isolates (Payne et al., 2005). The exact mode of action of these commercial biopesticides was not proven at the time of their commercial use, but from our work it is clear that most of their anti-damping-off control properties are derived from the production of cepacin at the rhizosphere of germinating crops (Figure 3B; Mullins et al., 2019).

With extensive specialised metabolite, genomic and taxonomic research on *Burkholderia* there has been renewed interest in repurposing these bacteria as biopesticides for several crops. This has included the use of new *Burkholderia* species or strains to control plant pathogens, such as *Botrytis cinerea* in grapevines (Esmaeel et al., 2020), *Fusarium oxysporum* in banana (Xu et al., 2020), *Sporisorium scitamineum* in sugarcane (Cui et al., 2020), and *Rhizoctonia cerealis* in wheat (An et al., 2022) via specialised metabolite production. In some cases, *Burkholderia gladioli* have also been found as endophytes in wild and ancient maize and shown to combat the fungal pathogen, *Sclerotinia homoeocarpa* (Shehata et al., 2016). Whereas *Burkholderia* sp. MSSP synthesizes 2-hydroxymethyl-chroman-4-one, demonstrating activity against *Pythium*, *Phytophthora* and *Sclerotinia* (Kang et al., 2004), and *B. ambifaria* strains inhibited phytopathogenic fungi through the emission of volatile organic compounds (Groenhagen et al., 2013). Alongside specialised metabolites, *B. gladioli* NGJ1 deploys a prophage tail-like protein secreted by a type III secretion system essential for mycophagy in *Rhizoctonia solani* (Swain et al., 2017).

*B. ambifaria* BCC0191 and *B. gladioli* BCC1697 also showed *in vitro* activity against the problematic wheat ‘take-all’ fungal pathogen (Palma-Guerrero et al., 2021), *Gaeumannomyces tritici* (Supplementary Figure S4). Recently, the mode of bacterial polyynes was demonstrated to inhibit a fungal-specific acetyl-CoA acetyltransferase in the first step of ergosterol biosynthesis (Lin et al., 2022). This indicates the potential use of polyyne-producing *Burkholderia* as biocontrol agents on a range of soil-borne fungal diseases that affect different crop species.

### 3.4. *Burkholderia ambifaria* cepacin expression and colonisation at the rhizosphere

The comparative biological control experiments highlighted considerable variation in the biocontrol efficacy of the seven cepacin-producing *B. ambifaria* strains (3.1–50% mean pea survival rate; Table 2), with BCC0191 demonstrating the greatest protection against damping-off (Figure 3B). This led us to explore the potential reasons for intraspecies biocontrol variation, examining: (i) cepacin gene cluster expression at the rhizosphere, (ii) the rate of *B. ambifaria* root colonisation, and (iii) the persistence of the *B. ambifaria* strains within soil (a non-sterile potting mix) microcosms. Two *B. ambifaria* strains, BCC1259 and BCC1237, that performed poorly in biocontrol (Figure 3B), but exhibited similar bioactivity against *G. ultimum* on PEM (Figure 3A), were compared to the *B. ambifaria* biopesticide strain BCC0191. Both BCC1237 and BCC1259 exhibited biological control levels ≤12.5% in contrast to BCC0191 with a mean survival rate of 50.6% (Figure 3B and Table 2).

After 7 days of plant growth, *B. ambifaria* BCC0191 and BCC1237 exhibited similar root colonisation levels, with average counts of 4.6 × 10^4^ and 6.6 × 10^4^ colony forming units (cfu) root section^−1^, respectively (Supplementary Figure S5B). In comparison, strain BCC1259 showed a consistently lower colonisation, with an average of 1.2 × 10^4^ cfu root section^−1^ (Supplementary Figure S5B). Interestingly, cepacin production levels on PEM also showed that BCC1259 produced consistently lower amounts of the polyyne than both BCC0191 and BCC1237 (Supplementary Figure S5A). Variation in soil persistence was observed by comparing each strains’ viable count per gram of soil following a 7-day incubation within a soil microcosm (Supplementary Figure S5C). The less protective strains, *B. ambifaria* BCC1237 and BCC1259, had viable counts that were an average of 11.3% and 12.2%, respectively, of their initial inoculum, whereas the more bioprotective strain BCC0191 persisted in the soil over 7 days at a level of 27.6% of the initial inoculum. Overall, this demonstrated that BCC0191 can survive and compete in a mixed, non-sterile soil (potting mix) microbial community better than other strains of *B. ambifaria* (Supplementary Figure S5C). The induction and expression of the cepacin BGC by *B. ambifaria* directly in the rhizosphere was determined by RT-PCR targeting the desaturase gene *ccnN* (Mullins et al., 2019). This PCR method was applied to total RNA extracted from the rhizosphere following 3 days of plant growth. A RT-PCR amplicon correlating to the expression of the *ccnN* gene was observed in all three strains (Supplementary Figure S6) and demonstrated that the cepacin gene BGC was readily expressed by all *B. ambifaria* strains at the pea rhizosphere.

Overall, this comparative evaluation of phenotypic traits demonstrated that multiple factors, in addition to polyyne production, play a role in the biological control efficacy of *B. ambifaria* strains. Successful rhizosphere colonisation, and the ability of the bacterium to persist and compete with the soil microbial community are clearly important attributes for an effective biocontrol agent. It is probable that the combination of these factors along with other qualities, including production of other antimicrobials (Mullins et al., 2019; Webster et al., 2020a), allows *B. ambifaria* BCC0191 to be a more efficient biocontrol agent in the *G. ultimum* damping-off assay than other strains of *B. ambifaria*. Previously, it has been shown that many *Pseudomonas* species are effective biocontrol agents due to their ability to colonise the plant surface (spermosphere, rhizosphere and phyllosphere) and the endosphere (Lugtenberg et al., 2001). These Pseudomonads not only protect plants by producing bioactive metabolites but can also use plant exudates with high growth rates, allowing them to compete with other microorganisms for space and nutrients in the plant environment (Lugtenberg et al., 2001). For example, the biocontrol ability of *P. fluorescens* strain 54/96 in the control of *Pythium* damping-off disease is based on their capacity to colonise plant tissues, exhibit high growth rates and outcompete the pathogen for limited plant nutrients and infection sites (Ellis et al., 1999). Colonisation of plant roots by bacterial endophytes through crack entry between adjacent cells, during emergence of lateral roots is well established (Webster et al., 1998).

### 3.5. Summary and conclusion

Given the lack of studies on *Burkholderia* biocontrol agents since the EPA risk assessment moratorium in 1999, there has been a comparative dearth of investigation into the mode of action and efficacy of strains within this genus compared to *Pseudomonas* and *Bacillus* where multiple strains and metabolites have been studied (Dimkić et al., 2022). The exploration of *Burkholderia* species has rightly focused on their pathogenic traits over the past two decades, however, multiple studies are now beginning to uncover the mechanisms and specialised metabolites that underpin their biological control proficiency (Mullins et al., 2019; Murata et al., 2021). However, it was unknown whether other *Burkholderia* strains or bacterial species encoding cepacin, caryoynencin and other polyyne BGCs were capable of similar protective biopesticidal roles. Our strain panel represented nine species and four polyyne BGCs, and *in vitro* microbial antagonisms was systematically compared to their efficacy in a pea biological control model with *G. ultimum* damping-off disease.

Considerable differences were observed in biocontrol proficiency between evolutionarily close species of *B. ambifaria* and *B. vietnamiensis* despite equivalent *in vitro* microbial antagonism and cepacin production (Figures 2, 3). In contrast to its widely characterised plant and human pathogenic nature (Jones et al., 2021), *B. gladioli* was uniquely shown to be an efficacious anti-damping off agent when interacted with germinating plants (Figure 3B), with the polyyne caryoynencin demonstrated to be a key metabolite in mediating *G. ultimum* biocontrol. Moreover, despite the protective *B. gladioli* strain being shown to be capable of producing broad-host range toxins such as toxoflavin and bongkrekic acid *in vitro*, these metabolites were suppressed on the biomimetic PEM medium, and the excellent pea survival rate suggested that toxic metabolites or pathogenic factors were not being deployed during the pea rhizosphere colonisation. Overall, the protective phenotype of caryoynencin, associated with rhizosphere colonisation of germinating peas exhibited by *B. gladioli* and *B. plantarii* in the biocontrol model, contrasts to the historical perspective of these bacteria as plant pathogens (Maeda et al., 2006; Jones et al., 2021). The beneficial interactions observed in this study suggests that the plant pathogenic lifestyle of *B. gladioli* and *B. plantarii* is likely host specific and opportunistic dependent on multiple factors such as host age and damage.

Our study demonstrated that certain *Burkholderia* strains with the capacity to produce polyynes act as optimal biological control agents for damping off-disease when applied as a seed coat to peas. This aligns to their successful commercial use and sheds light on their mechanism of action as biopesticides capable of protecting peas, maize, and other crops species (Parke and Gurian-Sherman, 2001). Interestingly, *Pseudomonas protegens* lacked noticeable biocontrol activity against *G. ultimum* on peas despite *in vitro* antagonism (Figure 3) and the observed protection mediated by protegencin in the biocontrol of *G. ultimum* in a cucumber model (Murata et al., 2021). These differences suggest host-specific interactions, as well as abilities such as rhizosphere colonisation rate, persistence, antimicrobial arsenal, and the ability to compete within the soil-associated microbial community play additional roles in successful biological control. Uncovering these factors and utilising them towards sustainable control of crop damping-off diseases is vital for food security going forward.

## Data availability statement

The original contributions presented in the study are included in the article/Supplementary material, further inquiries can be directed to the corresponding authors.

## Author contributions

GW, AM, and EM: conceptualisation, project administration, and writing – original draft. GW and AM: data curation, formal analysis, methodology, and software. EM: funding acquisition, resources, and supervision. GW, AM, and YP: investigation and visualisation. GW, AM, YP, and EM: validation and writing – review and editing. All authors contributed to the article and approved the submitted version.

## Funding

This research was funded by Biotechnology and Biological Sciences Research Council (BBSRC) and grant BB/S007652/1 (EM, AM, and GW). YP was supported by the UKRI-BBSRC South West Biosciences Doctoral Training Partnership (SWBio DTP; award BV19107109).

## Conflict of interest

The authors declare that the research was conducted in the absence of any commercial or financial relationships that could be construed as a potential conflict of interest.

## Publisher’s note

All claims expressed in this article are solely those of the authors and do not necessarily represent those of their affiliated organizations, or those of the publisher, the editors and the reviewers. Any product that may be evaluated in this article, or claim that may be made by its manufacturer, is not guaranteed or endorsed by the publisher.

## Footnotes

^1^ www.waters.com

^2^ www.lifeasible.com

## References

[ref1] Alcala A. V. C. Paulitz T. C. Schroeder K. L. Porter L. D. Derie M. L. Du Toit L. J. (2016). *Pythium* species associated with damping-off of pea in certified organic fields in the Columbia basin of central Washington. Plant Dis. 100, 916–925. doi: 10.1094/PDIS-07-15-0774-RE, PMID: 30686151

[ref2] An C. Ma S. Liu C. Ding H. Xue W. (2022). *Burkholderia ambifaria* XN08: a plant growth-promoting endophytic bacterium with biocontrol potential against sharp eyespot in wheat. Front. Microbiol. 13:906724. doi: 10.3389/fmicb.2022.906724, PMID: 35966702 PMC9368319

[ref3] Anwar M. Kasper A. Steck A. R. Schier J. G. (2017). Bongkrekic acid - a review of a lesser-known mitochondrial toxin. J. Med. Toxicol. 13, 173–179. doi: 10.1007/s13181-016-0577-1, PMID: 28105575 PMC5440313

[ref4] Azegami K. Nishiyama K. Watanabe Y. Kadota I. Ohuchi A. Fukazawa C. (1987). *Pseudomonas plantarii* sp. nov., the causal agent of rice seedling blight. Int. J. Syst. Bacteriol. 37, 144–152. doi: 10.1099/00207713-37-2-144

[ref5] Bach E. Passaglia L. M. P. Jiao J. Gross H. (2021). *Burkholderia* in the genomic era: from taxonomy to the discovery of new antimicrobial secondary metabolites. Crit. Rev. Microbiol. 48, 121–160. doi: 10.1080/1040841X.2021.1946009 34346791

[ref6] Baldwin A. Mahenthiralingam E. Drevinek P. Vandamme P. Govan J. R. Waine D. J.. (2007). Environmental *Burkholderia cepacia* complex isolates from human infections. Emerg. Infect. Dis. 13, 458–461. doi: 10.3201/eid1303.060403, PMID: 17552100 PMC2725883

[ref7] Bardin S. D. Huang H. C. Liu L. Yanke L. J. (2003). Control, by microbial seed treatment, of damping off caused by *Pythium* sp. on canola, safflower, dry pea, and sugar beet. Can. J. Plant Pathol. 25, 268–275. doi: 10.1080/07060660309507079

[ref8] Burkholder W. H. (1942). Three bacterial plant pathogens: *Phytomonas earyophylli* sp.n., *Phytomonas alliicola* sp.n., and *Phytomonas manihotis* (Arthaud-Berthet et Sondar) Viégas. Phytopathology 32, 141–149.

[ref9] Coenye T. Mahenthiralingam E. Henry D. Lipuma J. J. Laevens S. Gillis M.. (2001). *Burkholderia ambifaria* sp. nov., a novel member of the *Burkholderia cepacia* complex including biocontrol and cystic fibrosis-related isolates. Int. J. Syst. Evol. Microbiol. 51, 1481–1490. doi: 10.1099/00207713-51-4-1481 11491349

[ref10] Cui G. Yin K. Lin N. Liang M. Huang C. Chang C.. (2020). *Burkholderia gladioli* CGB10: a novel strain biocontrolling the sugarcane smut disease. Microorganisms 8:1943. doi: 10.3390/microorganisms8121943, PMID: 33297590 PMC7762381

[ref11] De Boer W. Leveau J. H. J. Kowalchuk G. A. Gunnewiek P. J. A. K. Abeln E. C. A. Figge M. J.. (2004). *Collimonas fungivorans* gen. nov., sp. nov., a chitinolytic soil bacterium with the ability to grow on living fungal hyphae. Int. J. Syst. Evol. Microbiol. 54, 857–864. doi: 10.1099/ijs.0.02920-0 15143036

[ref12] Dimkić I. Janakiev T. Petrović M. Degrassi G. Fira D. (2022). Plant-associated *Bacillus* and *Pseudomonas* antimicrobial activities in plant disease suppression via biological control mechanisms - a review. Physiol. Mol. Plant Pathol. 117:101754. doi: 10.1016/j.pmpp.2021.101754

[ref13] Dose B. Niehs S. P. Scherlach K. Shahda S. Flórez L. V. Kaltenpoth M.. (2021). Biosynthesis of sinapigladioside, an antifungal isothiocyanate from *Burkholderia* symbionts. Chembiochem 22, 1920–1924. doi: 10.1002/cbic.202100089, PMID: 33739557 PMC8252389

[ref14] Eberl L. Vandamme P. (2016). Members of the genus *Burkholderia*: good and bad guys. F1000Res 5:1007. doi: 10.12688/f1000research.8221.1, PMID: 27303639 PMC4882756

[ref15] El-Banna N. Winkelmann G. (1998). Pyrrolnitrin from *Burkholderia cepacia*: antibiotic activity against fungi and novel activities against streptomycetes. J. Appl. Microbiol. 85, 69–78. doi: 10.1046/j.1365-2672.1998.00473.x, PMID: 9721657

[ref16] Ellis R. J. Timms-Wilson T. M. Beringer J. E. Rhodes D. Renwick A. Stevenson L.. (1999). Ecological basis for biocontrol of damping-off disease by *Pseudomonas fluorescens* 54/96. J. Appl. Microbiol. 87, 454–463. doi: 10.1046/j.1365-2672.1999.00851.x, PMID: 10540249

[ref17] Esmaeel Q. Jacquard C. Sanchez L. Clément C. Ait Barka E. (2020). The mode of action of plant associated *Burkholderia* against grey mould disease in grapevine revealed through traits and genomic analyses. Sci. Rep. 10:19393. doi: 10.1038/s41598-020-76483-7, PMID: 33173115 PMC7655954

[ref18] Fira D. Dimkić I. Berić T. Lozo J. Stanković S. (2018). Biological control of plant pathogens by *Bacillus* species. J. Biotechnol. 285, 44–55. doi: 10.1016/j.jbiotec.2018.07.044, PMID: 30172784

[ref19] Flórez L. V. Scherlach K. Gaube P. Ross C. Sitte E. Hermes C.. (2017). Antibiotic-producing symbionts dynamically transition between plant pathogenicity and insect-defensive mutualism. Nat. Commun. 8:15172. doi: 10.1038/ncomms15172, PMID: 28452358 PMC5414355

[ref20] Fritsche K. Van Den Berg M. De Boer W. Van Beek T. A. Raaijmakers J. M. Van Veen J. A.. (2014). Biosynthetic genes and activity spectrum of antifungal polyynes from *Collimonas fungivorans* Ter331. Environ. Microbiol. 16, 1334–1345. doi: 10.1111/1462-2920.12440, PMID: 24588891

[ref21] Furuya N. Iiyama K. Shiozaki N. Matsuyama N. (1997). Phytotoxin produced by *Burkholderia gladioli*. J. Fac. Agric. Kyushu Univ. 42, 33–37. doi: 10.5109/24188

[ref22] Gilchrist C. L. M. Chooi Y.-H. (2021). Clinker & clustermap.js: automatic generation of gene cluster comparison figures. Bioinformatics 37, 2473–2475. doi: 10.1093/bioinformatics/btab007 33459763

[ref23] Gillis M. Van Van T. Bardin R. Goor M. Hebbar P. Willems A.. (1995). Polyphasic taxonomy in the genus *Burkholderia* leading to an emended description of the genus and proposition of *Burkholderia vietnamiensis* sp. nov. for N2-fixing isolates from rice in Vietnam. Int. J. Syst. Bacteriol. 45, 274–289. doi: 10.1099/00207713-45-2-274

[ref24] Gravel V. Martinez C. Antoun H. Tweddell R. J. (2005). Antagonist microorganisms with the ability to control *Pythium* damping-off of tomato seeds in rockwool. BioControl 50, 771–786. doi: 10.1007/s10526-005-1312-z

[ref25] Groenhagen U. Baumgartner R. Bailly A. Gardiner A. Eberl L. Schulz S.. (2013). Production of bioactive volatiles by different *Burkholderia ambifaria* strains. J. Chem. Ecol. 39, 892–906. doi: 10.1007/s10886-013-0315-y, PMID: 23832658

[ref26] Guo H. Roman D. Beemelmanns C. (2019). Tropolone natural products. Nat. Prod. Rep. 36, 1137–1155. doi: 10.1039/C8NP00078F 30556819

[ref27] Hendrix F. F. Campbell W. A. (1973). Pythiums as plant pathogens. Annu. Rev. Phytopathol. 11, 77–98. doi: 10.1146/annurev.py.11.090173.000453

[ref28] Hotter V. Zopf D. Kim H. J. Silge A. Schmitt M. Aiyar P.. (2021). A polyyne toxin produced by an antagonistic bacterium blinds and lyses a Chlamydomonad alga. Proc. Natl. Acad. Sci. 118:e2107695118. doi: 10.1073/pnas.2107695118, PMID: 34389682 PMC8379975

[ref29] Howell C. R. Stipanovic R. D. (1979). Control of *Rhizoctonia solani* on cotton seedlings with *Pseudomonas fluorescens* and with an antibiotic produced by the bacterium. Phytopathology 69, 480–482. doi: 10.1094/Phyto-69-480 40934404

[ref30] Hulot J. F. Hiller N. (2021). Exploring the benefits of biocontrol for sustainable agriculture – a literature review on biocontrol in light of the European Green Deal. Insti. Eur. Environ. Policy 1–42.

[ref31] Jassem A. N. Zlosnik J. E. A. Henry D. A. Hancock R. E. W. Ernst R. K. Speert D. P. (2011). *In vitro* susceptibility of *Burkholderia vietnamiensis* to aminoglycosides. Antimicrob. Agents Chemother. 55, 2256–2264. doi: 10.1128/AAC.01434-10, PMID: 21321142 PMC3088185

[ref32] Jenner M. Jian X. Dashti Y. Masschelein J. Hobson C. Roberts D. M.. (2019). An unusual *Burkholderia gladioli* double chain-initiating nonribosomal peptide synthetase assembles ‘fungal’ icosalide antibiotics. Chem. Sci. 10, 5489–5494. doi: 10.1039/C8SC04897E, PMID: 31293732 PMC6553374

[ref33] Jiao J. Du J. Frediansyah A. Jahanshah G. Gross H. (2020). Structure elucidation and biosynthetic locus of trinickiabactin from the plant pathogenic bacterium *Trinickia caryophylli*. J. Antibiot. 73, 28–34. doi: 10.1038/s41429-019-0246-0, PMID: 31605027

[ref34] Jin Y. Zhou J. Zhou J. Hu M. Zhang Q. Kong N.. (2020). Genome-based classification of *Burkholderia cepacia* complex provides new insight into its taxonomic status. Biol. Direct 15:6. doi: 10.1186/s13062-020-0258-5 32131884 PMC7057466

[ref35] Jones C. Webster G. Mullins A. J. Jenner M. Bull M. J. Dashti Y.. (2021). Kill and cure: genomic phylogeny and bioactivity of *Burkholderia gladioli* bacteria capable of pathogenic and beneficial lifestyles. Microb. Genomics 7:mgen000515. doi: 10.1099/mgen.0.000515 PMC8115902 33459584

[ref36] Kai K. Sogame M. Sakurai F. Nasu N. Fujita M. (2018). Collimonins A–D, unstable Polyynes with antifungal or pigmentation activities from the fungus-feeding Bacterium *Collimonas fungivorans* Ter331. Org. Lett. 20, 3536–3540. doi: 10.1021/acs.orglett.8b01311 29792438

[ref37] Kang J. G. Shin S. Y. Kim M. J. Bajpai V. Maheshwari D. K. Kang S. C. (2004). Isolation and anti-fungal activities of 2-Hydroxymethyl-chroman-4-one produced by *Burkholderia* sp. MSSP. Nihon Hōsenkin Gakkai shi 57, 726–731. doi: 10.7164/antibiotics.57.726, PMID: 15712667

[ref38] Katoh K. Standley D. M. (2013). MAFFT multiple sequence alignment software version 7: improvements in performance and usability. Mol. Biol. Evol. 30, 772–780. doi: 10.1093/molbev/mst010, PMID: 23329690 PMC3603318

[ref39] Kozlov A. M. Darriba D. Flouri T. Morel B. Stamatakis A. (2019). RAxML-NG: a fast, scalable and user-friendly tool for maximum likelihood phylogenetic inference. Bioinformatics 35, 4453–4455. doi: 10.1093/bioinformatics/btz305, PMID: 31070718 PMC6821337

[ref40] Kumar J. Ramlal A. Mallick D. Mishra V. (2021). An overview of some biopesticides and their importance in plant protection for commercial acceptance. Plan. Theory 10:1185. doi: 10.3390/plants10061185, PMID: 34200860 PMC8230470

[ref41] Kunakom S. Eustáquio A. S. (2019). *Burkholderia* as a source of natural products. J. Nat. Prod. 82, 2018–2037. doi: 10.1021/acs.jnatprod.8b01068, PMID: 31294966 PMC6871192

[ref42] Kusumi T. Ohtani I. Nishiyama K. Kakisawa H. (1987). Caryoynencins, potent antibiotics from a plant pathogen. Tetrahedron Lett. 28, 3981–3984. doi: 10.1016/S0040-4039(00)96437-2

[ref43] Lamichhane J. R. Dürr C. Schwanck A. A. Robin M.-H. Sarthou J.-P. Cellier V.. (2017). Integrated management of damping-off diseases. A review. Agronomie 37:10. doi: 10.1007/s13593-017-0417-y

[ref44] Li X. Li Y. Wang R. Wang Q. Lu L. (2019). Toxoflavin produced by *Burkholderia gladioli* from *Lycoris aurea* is a new broad-spectrum fungicide. Appl. Environ. Microbiol. 85:e00106-19. doi: 10.1128/AEM.00106-19 30824447 PMC6495751

[ref45] Lin C.-C. Hoo S. Y. Ma L.-T. Lin C. Huang K.-F. Ho Y.-N.. (2022). Integrated omics approach to unveil antifungal bacterial polyynes as acetyl-CoA acetyltransferase inhibitors. Commun. Biol. 5:454. doi: 10.1038/s42003-022-03409-6 35551233 PMC9098870

[ref46] Lipuma J. J. (2010). The changing microbial epidemiology in cistic fibrosis. Clin. Microbiol. Rev. 23, 299–323. doi: 10.1128/CMR.00068-09, PMID: 20375354 PMC2863368

[ref47] Loper J. E. Henkels M. D. Shaffer B. T. Valeriote F. A. Gross H. (2008). Isolation and identification of rhizoxin analogs from *Pseudomonas fluorescens* Pf-5 by using a genomic mining strategy. Appl. Environ. Microbiol. 74, 3085–3093. doi: 10.1128/AEM.02848-07, PMID: 18344330 PMC2394923

[ref48] Lugtenberg B. J. J. Dekkers L. Bloemberg G. V. (2001). Molecular determinants of rhizosphere colonization by *Pseudomonas*. Annu. Rev. Phytopathol. 39, 461–490. doi: 10.1146/annurev.phyto.39.1.461 11701873

[ref49] Maeda Y. Shinohara H. Kiba A. Ohnishi K. Furuya N. Kawamura Y.. (2006). Phylogenetic study and multiplex PCR-based detection of *Burkholderia plantarii*, *Burkholderia glumae* and *Burkholderia gladioli* using *gyrB* and *rpoD* sequences. Int. J. Syst. Evol. Microbiol. 56, 1031–1038. doi: 10.1099/ijs.0.64184-0, PMID: 16627650

[ref50] Mahenthiralingam E. Song L. Sass A. White J. Wilmot C. Marchbank A.. (2011). Enacyloxins are products of an unusual hybrid modular polyketide synthase encoded by a cryptic *Burkholderia ambifaria* genomic island. Chem. Biol. 18, 665–677. doi: 10.1016/j.chembiol.2011.01.020 21609847

[ref51] Mao W. Lewis J. A. Hebbar P. K. Lumsden R. D. (1997). Seed treatment with a fungal or a bacterial antagonist for reducing corn damping-off caused by species of *Pythium* and *Fusarium*. Plant Dis. 81, 450–454. doi: 10.1094/PDIS.1997.81.5.450 30861920

[ref52] Mullins A. J. Jones C. Bull M. J. Webster G. Parkhill J. Connor T. R.. (2020). Genomic assemblies of members of *Burkholderia* and related genera as a resource for natural product discovery. Microbiol. Resour. Announc. 9:e00485-20. doi: 10.1128/MRA.00485-20 33060263 PMC7561682

[ref53] Mullins A. J. Mahenthiralingam E. (2021). The hidden genomic diversity, specialized metabolite capacity, and revised taxonomy of *Burkholderia* sensu lato. Front. Microbiol. 12:726847. doi: 10.3389/fmicb.2021.726847, PMID: 34650530 PMC8506256

[ref54] Mullins A. J. Murray J. A. H. Bull M. J. Jenner M. Jones C. Webster G.. (2019). Genome mining identifies cepacin as a plant-protective metabolite of the biopesticidal bacterium *Burkholderia ambifaria*. Nat. Microbiol. 4, 996–1005. doi: 10.1038/s41564-019-0383-z, PMID: 30833726 PMC6544543

[ref55] Mullins A. J. Webster G. Kim H. J. Zhao J. Petrova Y. D. Ramming C. E.. (2021). Discovery of the *Pseudomonas* polyyne protegencin by a phylogeny-guided study of polyyne biosynthetic gene cluster diversity. mBio 12:e0071521. doi: 10.1128/mBio.00715-21, PMID: 34340549 PMC8406139

[ref56] Murata K. Suenaga M. Kai K. (2021). Genome mining discovery of protegenins A–D, bacterial polyynes involved in the antioomycete and biocontrol activities of *Pseudomonas protegens*. ACS Chem. Biol. 17, 3313–3320. doi: 10.1021/acschembio.1c00276 34015911

[ref57] Neidig N. Paul R. J. Scheu S. Jousset A. (2011). Secondary metabolites of *Pseudomonas fluorescens* CHA0 drive complex non-trophic interactions with bacterivorous nematodes. Microb. Ecol. 61, 853–859. doi: 10.1007/s00248-011-9821-z, PMID: 21360140 PMC3098371

[ref58] Nicolopoulou-Stamati P. Maipas S. Kotampasi C. Stamatis P. Hens L. (2016). Chemical pesticides and human health: the urgent need for a new concept in agriculture. Front. Public Health 4:148. doi: 10.3389/fpubh.2016.00148 27486573 PMC4947579

[ref59] Palma-Guerrero J. Chancellor T. Spong J. Canning G. Hammond J. McMillan V. E.. (2021). Take-all disease: new insights into an important wheat root pathogen. Trends Plant Sci. 26, 836–848. doi: 10.1016/j.tplants.2021.02.009, PMID: 33752966

[ref60] Parke J. L. Gurian-Sherman D. (2001). Diversity of the *Burkholderia cepacia* complex and implications for risk assessment of biological control strains. Annu. Rev. Phytopathol. 39, 225–258. doi: 10.1146/annurev.phyto.39.1.225 11701865

[ref61] Parker W. L. Rathnum M. L. Seiner V. Trejo W. H. Principe P. A. Sykes R. B. (1984). Cepacin A and cepacin B, two new antibiotics produced by *Pseudomonas cepacia*. J. Antibiot. 37, 431–440. doi: 10.7164/antibiotics.37.431, PMID: 6547430

[ref62] Paulsen I. T. Press C. M. Ravel J. Kobayashi D. Y. Myers G. S. A. Mavrodi D. V.. (2005). Complete genome sequence of the plant commensal *Pseudomonas fluorescens* Pf-5. Nat. Biotechnol. 23, 873–878. doi: 10.1038/nbt1110, PMID: 15980861 PMC7416659

[ref63] Payne G. W. Vandamme P. Morgan S. H. Lipuma J. J. Coenye T. Weightman A. J.. (2005). Development of a *recA* gene-based identification approach for the entire *Burkholderia* genus. Appl. Environ. Microbiol. 71, 3917–3927. doi: 10.1128/AEM.71.7.3917-3927.2005, PMID: 16000805 PMC1169057

[ref64] Petrova Y. D. Zhao J. Webster G. Mullins A. J. Williams K. Alswat A. S.. (2022). Cloning and expression of *Burkholderia* polyyne biosynthetic gene clusters in *Paraburkholderia* hosts provides a strategy for biopesticide development. Microb. Biotechnol. 15, 2547–2561. doi: 10.1111/1751-7915.14106 35829647 PMC9518984

[ref65] Philmus B. Shaffer B. T. Kidarsa T. A. Yan Q. Raaijmakers J. M. Begley T. P.. (2015). Investigations into the biosynthesis, regulation, and self-resistance of toxoflavin in *Pseudomonas protegens* Pf-5. Chembiochem 16, 1782–1790. doi: 10.1002/cbic.201500247, PMID: 26077901

[ref66] Punja Z. K. Yip R. (2003). Biological control of damping-off and root rot caused by *Pythium aphanidermatum* on greenhouse cucumbers. Can. J. Plant Pathol. 25, 411–417. doi: 10.1080/07060660309507098

[ref67] Ross C. Scherlach K. Kloss F. Hertweck C. (2014). The molecular basis of conjugated polyyne biosynthesis in phytopathogenic bacteria. Angew. Chem. Int. Ed. 53, 7794–7798. doi: 10.1002/anie.201403344, PMID: 24898429

[ref68] Shehata H. R. Lyons E. M. Jordan K. S. Raizada M. N. (2016). Bacterial endophytes from wild and ancient maize are able to suppress the fungal pathogen *Sclerotinia homoeocarpa*. J. Appl. Microbiol. 120, 756–769. doi: 10.1111/jam.13050, PMID: 26742658

[ref69] Song L. Jenner M. Masschelein J. Jones C. Bull M. J. Harris S. R.. (2017). Discovery and biosynthesis of gladiolin: a *Burkholderia gladioli* antibiotic with promising activity against *Mycobacterium tuberculosis*. J. Am. Chem. Soc. 139, 7974–7981. doi: 10.1021/jacs.7b03382 28528545

[ref70] Stutz E. W. Défago G. Kern H. (1986). Naturally occurring fluorescent pseudomonads involved in suppression of black root rot of tobacco. Phytopathology 76, 181–185. doi: 10.1094/Phyto-76-181

[ref71] Swain D. M. Yadav S. K. Tyagi I. Kumar R. Kumar R. Ghosh S.. (2017). A prophage tail-like protein is deployed by *Burkholderia* bacteria to feed on fungi. Nat. Commun. 8:404. doi: 10.1038/s41467-017-00529-0, PMID: 28864820 PMC5581363

[ref72] Uzuhashi S. Kakishima M. Tojo M. (2010). Phylogeny of the genus *Pythium* and description of new genera. Mycoscience 51, 337–365. doi: 10.1007/S10267-010-0046-7

[ref73] Vanlaere E. Lipuma J. J. Baldwin A. Henry D. De Brandt E. Mahenthiralingam E.. (2008). *Burkholderia latens* sp. nov., *Burkholderia diffusa* sp. nov., *Burkholderia arboris* sp. nov., *Burkholderia seminalis* sp. nov. and *Burkholderia metallica* sp. nov., novel species within the *Burkholderia cepacia* complex. Int. J. Syst. Evol. Microbiol. 58, 1580–1590. doi: 10.1099/ijs.0.65634-0 18599699

[ref74] Webster G. Jain V. Davey M. R. Gough C. Vasse J. Dénarié J.. (1998). The flavonoid naringenin stimulates the intercellular colonization of wheat roots by *Azorhizobioum caulinodans*. Plant Cell Environ. 21, 373–383. doi: 10.1046/j.1365-3040.1998.00278.x

[ref75] Webster G. Jones C. Mullins A. J. Mahenthiralingam E. (2020a). A rapid screening method for the detection of specialised metabolites from bacteria: induction and suppression of metabolites from *Burkholderia* species. J. Microbiol. Methods 178:106057. doi: 10.1016/j.mimet.2020.106057, PMID: 32941961 PMC7684528

[ref76] Webster G. Mullins A. J. Cunningham-Oakes E. Renganathan A. Aswathanarayan J. B. Mahenthiralingam E.. (2020b). Culturable diversity of bacterial endophytes associated with medicinal plants of the Western Ghats, India. FEMS Microbiol. Ecol. 96:fiaa147. doi: 10.1093/femsec/fiaa147 32710748 PMC7422900

[ref77] White J. Gilbert J. Hill G. Hill E. Huse S. M. Weightman A. J.. (2011). Culture-independent analysis of bacterial fuel contamination provides insight into the level of concordance with the standard industry practice of aerobic cultivation. Appl. Environ. Microbiol. 77, 4527–4538. doi: 10.1128/AEM.02317-10, PMID: 21602386 PMC3127687

[ref78] Xu Z. Wang M. Du J. Huang T. Liu J. Dong T.. (2020). Isolation of *Burkholderia* sp. HQB-1, a promising biocontrol bacteria to protect banana against Fusarium wilt through phenazine-1-carboxylic acid secretion. Front. Microbiol. 11:605152. doi: 10.3389/fmicb.2020.605152 33362750 PMC7758292

