# Supplementary material for: Polyyne-producing Burkholderia suppress Globisporangium ultimum damping-off disease of Pisum sativum (pea)
Source: Front Microbiol. 2023 Aug 25;14:1240206. doi: 10.3389/fmicb.2023.1240206 (PMC10485841; doi:10.3389/fmicb.2023.1240206)
Supplement: Supplementary file 1 [file Data_Sheet_1.docx]

**SUPPLEMENTAL MATERIAL**

**Polyyne-producing *Burkholderia* suppress *Globisporangium ultimum* damping-off disease of *Pisum sativum* (pea)**

Gordon Webster^1#^*, Alex J. Mullins^1^* Yoana D. Petrova^1^, and Eshwar Mahenthiralingam^1#^

^1^Microbiomes, Microbes and Informatics Group, Organisms and Environment Division, School of Biosciences, Cardiff University, Cardiff, CF10 3AX, United Kingdom.


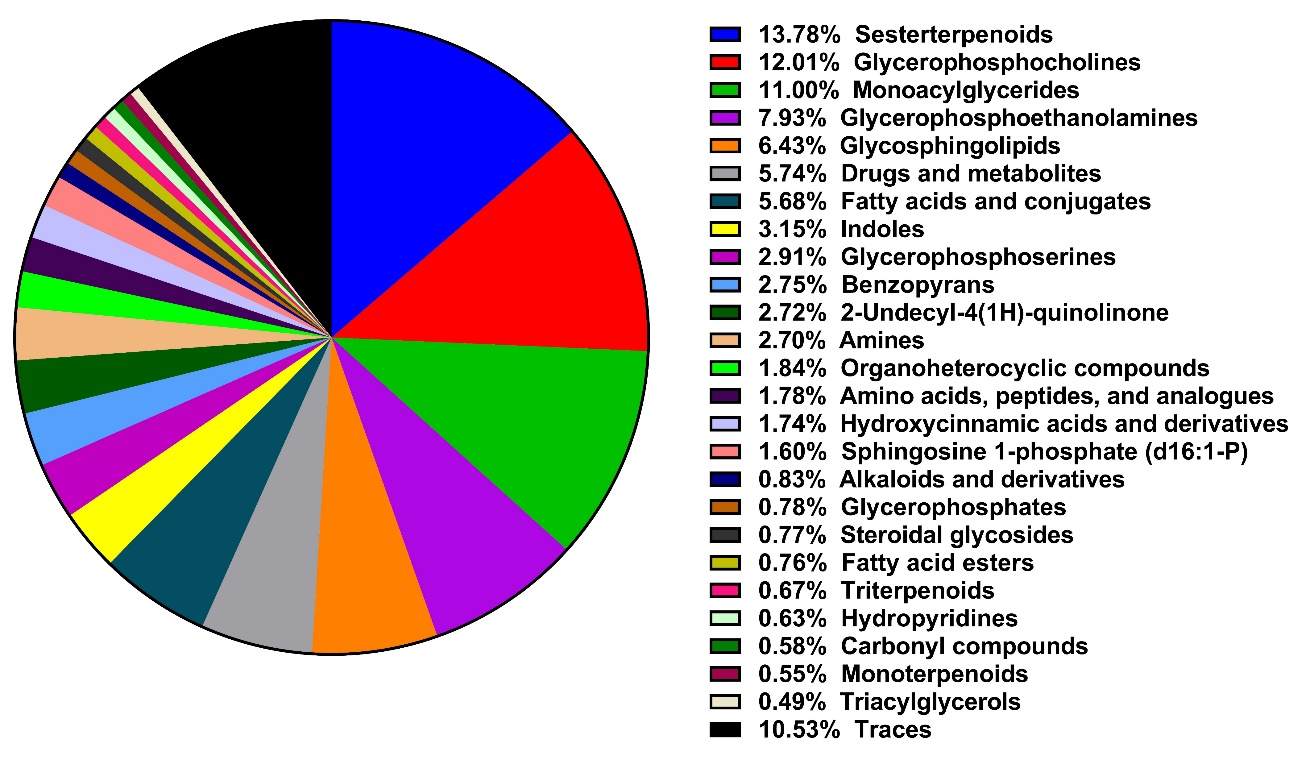


**Figure S1. Relative concentrations of compound sub-classes identified in pea seed exudate media (PEM) using liquid chromatography-mass spectrometry (HPLC-MS/MS).** Compounds identified are represented as a percentage of known metabolites identified in positive ion mode. Traces represent compounds with relative abundance <0.5%. Identification of known compounds was performed by Lifeasible analytical services ([www.lifeasible.com](http://www.lifeasible.com)). using the HMDB online database (<https://hmdb.ca/>).

**Figure S2. Examples of antagonism assays for screening polyyne-producing bacteria against a range of pathogens on BSMG and PEM.** (a) *Burkholderia ambifaria* BCC0191 against *Staphylococcus aureus*, *Pectobacterium carotovorum*, *Candida albicans*, and *Clavibacter michiganensis*. Distinct zones of clearing can be seen against *S. aureus* and *Ca. albicans*. (b) *Burkholderia gladioli* BCC1697 against *S. aureus*, *Pe. carotovorum*, *Ca. albicans*, and *Cl. michiganensis*. Distinct zones of clearing can be seen against *S. aureus*, *Ca. albicans*, and *Cl. michiganensis*. (c) *B. ambifaria* BCC0191 and *B. gladioli* BCC1697 against *Globisporangium ultimum.* Distinct antagonism by both *Burkholderia* species can be seen in the form of reduced *G. ultimum* growth compared with the control.

**Figure S3.** **Biocontrol efficacy of *Burkholderia ambifaria* BCC191 and *Burkholderia gladioli* BCC1697 compared with their respective polyyne BGC disrupted mutants**. (a) An example biological control assay is shown for each strain and their respective mutant. (b) The mean survival rate of the peas after 14 days is plotted for a total of n = 2 experiments. Gu = *G. ultimum* causal agent of damping off disease in peas.

**Figure S4.** ***In vitro* antagonism assay for *Burkholderia* polyyne-producers against the take-all fungus, *Gaeumannomyces tritici* on BSMG, PDA and seed exudate media.** (a) *B. ambifaria* BCC0191 and (b) *B. gladioli* BCC1697 against *G. tritici*. Distinct antagonism by both *Burkholderia* species (centre colony) can be seen in the form of reduced *G. tritici* growth. Seed exudate media was made from pea (left) and wheat (right) seeds.

**Figure S5. Comparison of cepacin production, root colonisation and soil persistence of *Burkholderia ambifaria* strains BCC0191, BCC1237 and BCC1259.** (a) Semi-quantitative comparison of cepacin production on pea exudate medium (PEM) by HPLC analysis. (b) Comparison of rhizosphere colonisation after 3 days determined by total viable count of *B. ambifaria* from the first 1-2 cm of root on BCSA. (c) Recoverable viable count (soil persistence) compared to initial inoculum of *B. ambifaria* strains in plant potting mix (soil) after 7 days incubation. % persistence = cell count at 7 days/ cell count at 0 days x 100.

**Figure S6. Reverse-transcription PCR for desaturase gene *ccnN* after 3 days of *B. ambifaria* colonisation on pea roots**. Lanes are as follows: M, 100 bp to 10 kbp marker (Ladder I; PCR Biosystems); lane 1, BCC0191 on filter paper; lane 2, BCC1237 on filter paper; lane 3, BCC1259 on filter paper; lane 4, BCC0191 on rhizosphere; lane 5, BCC1237 on rhizosphere; lane 6, BCC1259 on rhizosphere; lane 7, positive control (BCC0191 on BSMG); lane 8, negative control. RT-PCR product is 514 bp.
