# Supplementary figures and images for: Polyyne-producing Burkholderia suppress Globisporangium ultimum damping-off disease of Pisum sativum (pea)
Source: Front Microbiol. 2023 Aug 25;14:1240206. doi: 10.3389/fmicb.2023.1240206 (PMC10485841; doi:10.3389/fmicb.2023.1240206)

## Slide 1
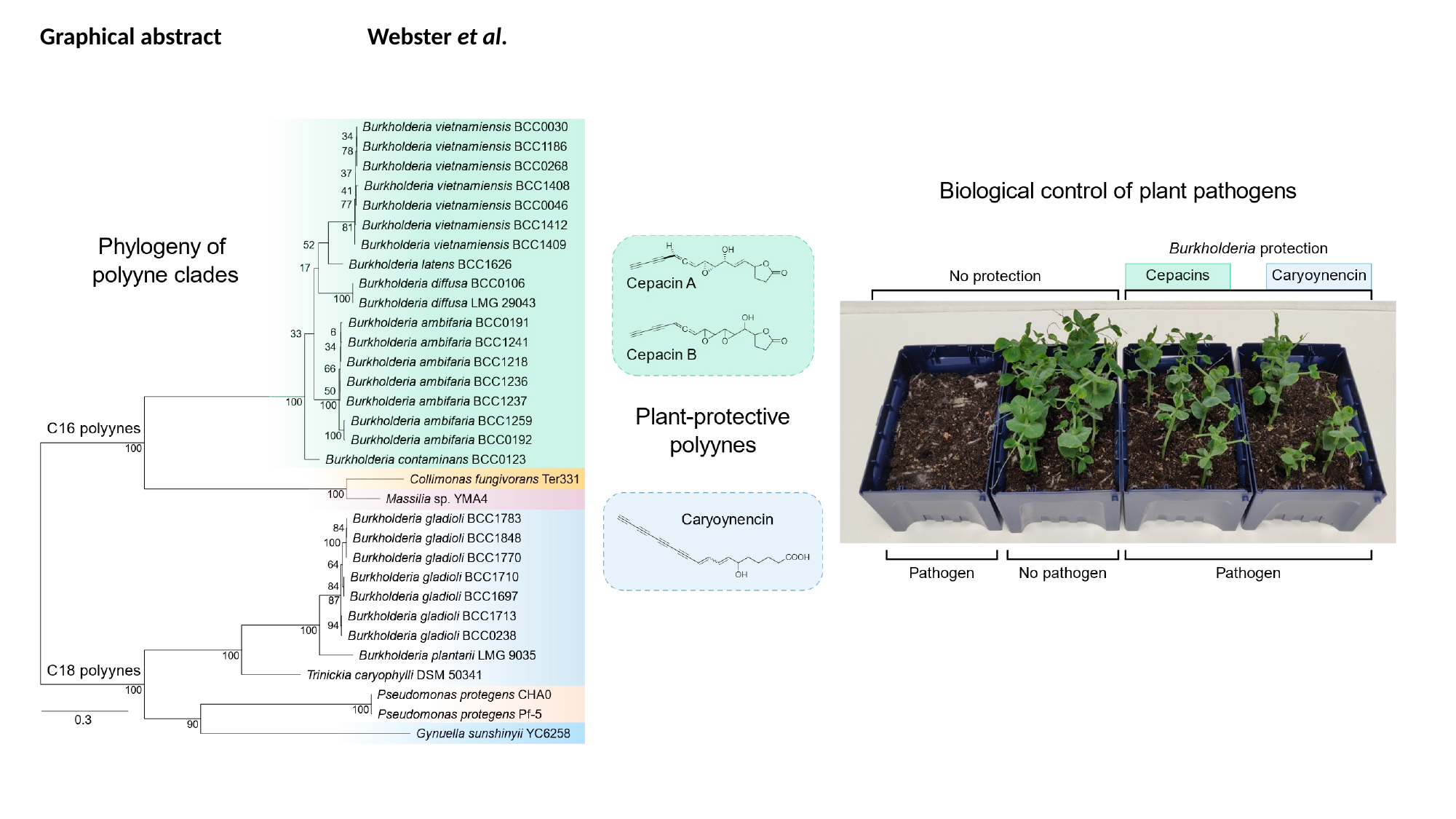

Graphical abstract		Webster et al.

Supplement: Supplementary file 2 [file Presentation_1.PPTX]
